# Epidemiological study of leptospiral interaction in bovine farms in rural areas of Colombia: A One Health approach

**DOI:** 10.1371/journal.pntd.0014231

**Published:** 2026-05-06

**Authors:** Sara Patiño-Gómez, Luisa F. Naranjo-Vargas, Daniel C. Aguirre-Acevedo, Néstor Jaime Aguirre-Ramírez, Elsio A. Wunder Jr, Felipe de Oliveira, Samanta C. das Chagas-Xavier, Juan C. Quintero-Vélez

**Affiliations:** 1 Grupo de Epidemiología, Universidad de Antioquia, Medellín, Antioquia, Colombia; 2 Department of Pathobiology and Veterinary Science, University of Connecticut, Storrs, Connecticut, United States of America; 3 Grupo de Investigación en Ciencias Veterinarias CENTAURO, Universidad de Antioquia, Medellín, Colombia; 4 Facultad de Medicina, Universidad de Antioquia, Medellín, Colombia; 5 Grupo de Investigación GeoLimna, Universidad de Antioquia, Medellín, Antioquia, Colombia; 6 Gonçalo Moniz Institute, Oswaldo Cruz Foundation, Salvador, Brazil; 7 Laboratório de Biologia de Tripanosomatídeos, Instituto Oswaldo Cruz, Rio de Janeiro, Brazil; 8 Grupo Programa de Estudio y Control de Enfermedades Tropicales (PECET), Universidad de Antioquia, Medellín, Colombia; Faculty of Medicine and Health Sciences, Universiti Putra Malaysia, MALAYSIA

## Abstract

**Background:**

*Leptospira* are zoonotic agents with a complex transmission cycle that affects low-income and impoverished populations and causes significant economic losses in livestock.

**Objective:**

To evaluate the interaction between people, animals, and the environment related to *Leptospira* infection in bovine farms in Urabá, Antioquia.

**Methods:**

An exploratory cross-sectional study was conducted on cattle farms in Urabá, Antioquia. The proportion of pathogenic *Leptospira* infection in bovine and canine urine and environmental contamination in water and soil samples was estimated using molecular assays. Additionally, *Leptospira* seropositivity in humans, cattle, and canines was determined using the microagglutination test (MAT). Evaluation of composition characteristics of landscape was done and potential flooding areas were estimated. The domestic animals and human populations were characterized through descriptive analysis using productive and reproductive data and sociodemographic information, respectively. Then, associations between seropositivity/infection, antibody titers, *Leptospira* serogroups/species in cattle, canines, and humans, and productive, farms and landscape variables we explored using a mixed-data factor analysis.

**Results:**

The proportion of seropositivity in cattle wa 76.9% (380/494). The most frequent serogroups on MAT were Mini, Tarassovi, Ballum, and Sejroe. In addition, molecular analysis indicated an infection rate of 4.0% (20/494) of the species *L. borgpetersenii* in cattle*.* Seropositivity in humans was 4.1% (3/73), with serogroups Icterohaemorrhagiae, Autumnalis, and Sejroe. Thirty-three percent (5/15) of dogs were seropositive for serogroups Canicola, Icterohaemorrhagiae, Ballum, and Autumnalis. The presence of *L. tipperaryensis* was detected in water and species *L. weilii* and *L. cinconiae* in soil. Evidence of high exposure to Leptospira was found in cattle. An association was also found between the serogroups circulating in humans and dogs (Autumnalis) and in humans and cattle (Sejroe), as well as forest fragmentation.

**Conclusions:**

The importance of addressing the epidemiology of *Leptospira* infection from a comprehensive One Health approach is highlighted.

## Author summary

Leptospirosis is a neglected zoonotic disease with a global distribution, particularly affecting vulnerable populations in tropical and subtropical regions. This study approaches leptospirosis from a One Health perspective, recognizing the interconnectedness of human, animal, and environmental health. By integrating data from landscape, bovine farms, domestic animals, people, and environmental sources such as water and soil, this research provides a comprehensive understanding of the transmission dynamics of *Leptospira* in rural livestock systems. The findings contribute to the growing body of knowledge on emerging and re-emerging infectious diseases and highlight the need for integrated surveillance in marginalized regions. This approach is particularly valuable in areas with limited access to healthcare, veterinary services, and environmental monitoring, where the risk of transmission between species and ecosystems is heightened. The study underscores the importance of multidisciplinary efforts to address zoonotic threats and inform public health strategies aimed at prevention, early detection, and leptospirosis control.

## Introduction

The World Health Organization [1] defines the One Health approach as an integrated and unifying strategy whose objective is to sustainably optimize the health of people, animals, and ecosystems. Within this perspective, one of its main objectives is the prevention and control of emerging, re-emerging, and neglected infectious diseases [2]. In this sense, more than 50% of emerging infectious diseases in humans have a zoonotic origin, such as rabies, salmonellosis, West Nile virus, and leptospirosis [3].

Leptospirosis is a zoonotic and neglected disease with worldwide distribution, caused by infection of the pathogenic species of the *Leptospira* genus [4]. This disease is endemic in tropical regions, and outbreaks are common after periods of rain and flooding [5]. The infection occurs through direct or indirect contact with the urine or environmental sources contaminated with the urine of infected animals, especially cattle, dogs, and rodents, which act as reservoirs and spreaders of the pathogen [6]. In the livestock context, significant economic losses due to abortions, stillbirths, and reproductive disorders are caused by leptospirosis [7,8]. *Leptospira* infection presents a complex life cycle due to the diversity of hosts, *Leptospira* species, pathogenic serogroups, and different sources of infection in domestic, wild, and synanthropic animals, as well as in humans [9].

Leptospirosis in humans is primarily linked to poverty and poor sanitation [5], but occupational exposure, such as farmers, veterinarians, and sanitation workers, and recreational settings through contact with contaminated water could be a risk condition by acquiring the infection [10]. The incidence of leptospirosis is higher in rural areas and communities with unmet basic needs. Factors such as inadequate waste management, the presence of rodents, and limited access to basic sanitation increase exposure to leptospires [5]. Other conditions that favor the circulation of *Leptospira* include high humidity, waterlogged soils, bodies of water, loss of forest cover, and fragmented landscapes [11].

The Urabá region of Colombia has multiple factors that facilitate exposure to *Leptospira* in humans and domestic animals such as sociodemographic and ecological aspect linked to high poverty rates, with over 50% of the population living in rural areas, and an economy based on agriculture, livestock, and fishing [12]. Additionally, this region presents a high prospective change of landscapes and anthropization.

In this sense, bovine farms can play a central role in the persistence and spread of pathogens, especially in relation to landscape transformation caused by expanding pastures. This transformation decreases species diversity and increases close contact between humans, domestic animals, and wildlife [13]. These factors together create an ecological and social environment that facilitates the circulation of pathogenic *Leptospira* between animals, humans, and the environment. These elements make Urabá a key territory for studying *Leptospira* transmission dynamics from a One Health perspective. The overall objective of this study was to evaluate the interaction between people, animals, and the environment related to *Leptospira* infection in bovine farms from the Urabá region.

## Materials and methods

### Ethics statement

The Committee for Animal Experimentation and Committee of Ethics in Research of the University of Antioquia approved the research protocol for this study (meetings held on February 7 and June 23, 2023).

### Study design

**Fig 1:**
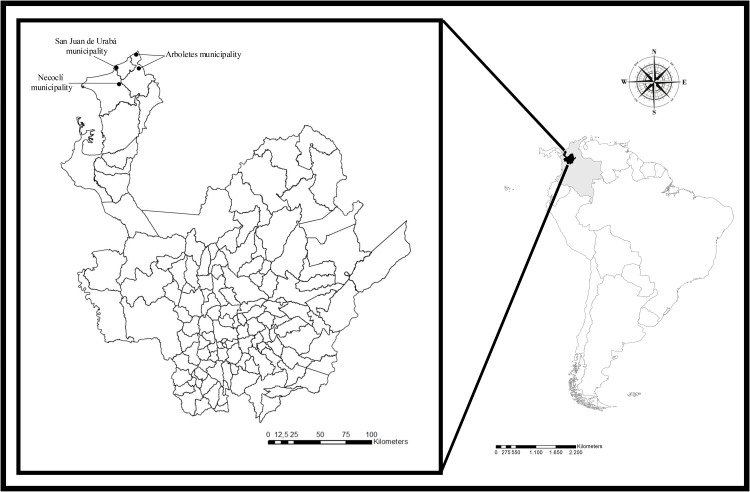
Geographic location of the municipalities surveyed in the study. Black dots show the municipalities where the study was conducted. The map was created in QGIS software using only openly licensed datasets. **Data Sources and References:** The base map layers used in the figure were obtained from publicly available sources compatible with CC BY 4.0 licensing. South America country boundaries were downloaded from Natural Earth (public domain): https://www.naturalearthdata.com/downloads/10m-cultural-vectors/. Colombia administrative boundaries (including Antioquia) were obtained from SimpleMaps under a Creative Commons Attribution 4.0 license (CC BY 4.0): https://simplemaps.com/gis/country/co.

From November to December 2023, an exploratory cross-sectional study was conducted on livestock farms located in the municipalities of Arboletes (8°51′0.98″ N, 76°25′36.91″ W), Necoclí (8°25′37.68″ N, 76°47′15.50″ W), and San Juan de Urabá (8°45′38.73″ N, 76°31′38.23″ W), in the Urabá region of Antioquia (Fig 1). Cattle farms dedicated to the breeding and rearing of beef cattle, with complete records of productive and reproductive data, and whose owners and managers agreed to participate in the study and signed the informed consent form, were included in the study. The inclusion criteria included cattle of any breed, age group, and productive status. Animals that were not suitable for handling due to veterinary medical criteria and areas with the presence of illegal groups were excluded.

For each farm enrolled, people who lived (housewives, students, among others) or worked (veterinarians, animal husbandry, cowboys, day laborers, or other workers) who were present at the time of the farm visit and who wished to participate and signed the informed consent and informed assent forms were included in the study. There were no exclusion criteria based on age, gender or ethnicity.

Additionally, all healthy canines living on each enrolled farm were included in the study. Finally, easily accessible soil and natural water sources used for animal consumption and human supply, located in different areas of the farms and near to their main facilities, were taken into account.

### Sampling and sample design

A non-probabilistic sampling was conducted to select seven farms, as these were the only farms that both met the inclusion criteria and expressed willingness and availability to participate in the study. Within these farms, bovines, canines, people, water and soil sources were included (Fig 1). Four farms were located in the municipality of Arboletes, one in San Juan de Urabá, one in Necoclí, and the remaining one between the latter two municipalities. Bovines were selected based on the annual vaccination campaigns against brucellosis and foot-and-mouth disease.

### Collection of sociodemographic information

Epidemiological surveys were designed to collect information on individuals’ sociodemographic characteristics and housing conditions. Prior to questionnaire design, characteristics with plausible relevance to *Leptospira* transmission in productive farm settings were evaluated to guide the selection of variables of interest. The questionnaires were then developed and pilot-tested in a rural area near Medellín among residents and workers of a dairy farm to assess clarity, comprehension, and the time required to complete the instruments.

Information related to age in years, gender, time living in the area, level of education, current and previous occupations, and place of occupation were collected. Also, the survey asked about potential conditions associated with *Leptospira* infection, such as contact with animal urine, placentas, or newborn animals, contact with animal water or food, contact with streams, lakes, ravines, contact with sewage, and the use of personal protective measures such as gloves and handwashing. Information related to practices such as walking barefoot and bathing or drinking water from natural water sources were considered.

A housing survey was completed by the head of the households. This survey asked about flooring, wall, roof materials, peridomiciliary characteristics (10 m around the house) (presence of bushes, trees, grasses, corn cultivation, cassava cultivation, and tomato cultivation) and presence of domestic (canines, felines, poultry, turkeys, pigs, horses, donkeys, and mules), synanthropic and wild animals (rodents and opossums), as well as practices related to risk factors for tropical infectious diseases (such as wastewater management, drinking water, coexistence with animals, and sanitation conditions inside and around the home). Preventive measures, such as the presence of ditches or barriers against potential flooding, rodent control, proper waste disposal, and drinking water treatment were also surveyed.

### Collection of information on bovines and canines

Productive and reproductive information from bovines were collected by a veterinarian using records generated by Ganadero Software, which was routinely used on each of the participating farms. Reproductive stage, age in years, breed, age at first breeding (months), age at first service (months), age at first calving (months), number of births, calving interval (days), open days, services per conception, body condition (score from 1 to 5), weaning weight (Kg), daily weight gain (gr), and reproductive diseases (abortions, stillbirths, mummified fetuses, and fetal resorption) were surveyed. Information on the zootechnical management practices used on each farm, including predominant pasture types, feeding methods, leptospirosis vaccination, pasture rotation intervals, and the presence of domestic and synanthropic animals in pastures were also considered.

For all canines in the study, information related to sex, age in years, breed, origin, body condition, vaccination status against *Leptospira*, internal deworming, and potential exposure to conditions that favor *Leptospira* infection, such as close contact with rodents and cattle, were surveyed.

### Analysis of vegetation cover and metrics in landscapes

Landscape information for each selected farm was captured using an unmanned aerial vehicle (UAV) DJI Mavic 3 Multispectral with automated flyovers at 120 m. The geographical boundaries of each farm were provided by their managers and all images obtained by drone were analyzed using Agisoft Metashape 1.8 software to perform a photogrammetric analysis and generate orthomosaics and digital elevation models (DEM).

The orthomosaics were integrated into the QGIS 3.40 software and an initial manual classification of the terrain was generated using the Semi-Automatic Classification Plugin (SCP). Five land use cover classes were included: (i) pastures or forage; (ii) forest or dense vegetation; (iii) water bodies (natural and artificial); (iv) built-up areas (houses, stables, roads, and buildings associated with agricultural production); and (v) crop cultivation (corn, bananas, plantains, and cassava, among others).

Subsequently, automatic classification using Python version 3.9 was carried out. The samples were divided into two sets; 80% for training and 20% for testing the models. The pixels from the training polygons were extracted and used to construct the classification model based on the Random Forest algorithm. After training models, pixels from each polygon were applied to estimate the classification accuracy. These results were then used to evaluate the models’ performance and their adequate representation of the classes in each image. Finally, the best-performing model was used to classify each image generated on each farm.

The performance of the Random Forest model was evaluated using a confusion matrix (S1 Table). Based on the classification results, a table to compare the reference data (samples) with the corresponding actual labels (predicted labels) was created. This analysis allowed to quantify the hits and errors per pixel. The evaluation metrics precision, recall, and F1 score were then calculated from the confusion matrix, enabling a detailed analysis of the classifier’s performance in relation to each mapped class.

After classification, the resulting images were subjected to a mean filter with a 9 × 9 window to smooth and adjust the classified pixels. This technique reduces noise, eliminates isolated pixels, and promotes greater spatial coherence between image elements, resulting in more accurate, visually homogeneous segmentation. The filter was applied using Python version 3.9.

Additionally, the classified images were analyzed using a Python version 3.9 algorithm with the PyLandStats library to quantify landscape metrics. The relevant metrics for the structural characterization of the landscape as total area (hectares [ha]), landscape proportion (%), number of patches, patch density (patches per 100 hectares [ha]), largest patch index (%), total edge (meters [m]), edge density (meters per hectare [m/ha]), and landscape shape index were extracted [14]. The results were exported in CSV format, which allows for a detailed quantitative description of the spatial configuration of the classes and facilitates the assessment of fragmentation, connectivity, and the landscape.

The DEMs were integrated into the QGIS 3.40 software to analyze the Topographic Wetness Index (TWI). This index was then calculated using the r.fill.dir and r.watershed algorithms to generate DEMs without depressions and TWI, respectively. A categorized TWI was calculated in each farm taking into account TWI ≥ 10 (floodable) and TWI < 10 (not floodable) to measure the total area with potential floodable. The TWIs were adjusted for the size of each farm, calculating floodable area respect (categorized TWI) the total area of each farm.

### Evaluation of seropositivity against Leptospira

Blood samples from humans, bovines and canines were collected in tubes containing clot retraction gel to facilitate serum collection and were kept refrigerated (4°C) until centrifugation. The serum obtained was then stored at -80°C until processing. Afterwards, the serum was taken to perform the microagglutination test (MAT) and determine seropositivity against *Leptospira*. The MAT was performed at the laboratory of the University of Connecticut using a panel of thirty-three strains, representing 24 serogroups and 12 species of the genus *Leptospira*. For bovines, to select the antigens for serum samples testing, a proportional random selection of 10% of total serum samples was performed to select the panel of strains for analyzing all samples. After the evaluation, a final of 15 antigens was selected to be tested with all bovine sera. Finally, all serum samples from humans and canines were processed using all 33 strains (Table 1).

**Table 1:** Panel of *Leptospira* strains used for the MAT in domestic animals and humans.

No.	Species	Serogroup	Serovar	Strain
1	*L. interrogans*	Djasiman	Djasiman	Djasiman
**2**	***L. interrogans***	**Icterohaemorrhagiae**	**Icterohaemorrhagiae**	**RGA**
**3**	***L. interrogans***	**Icterohaemorrhagiae**	**Copenhageni**	**M 20**
4	*L.weilii*	Javanica	Coxi	Cox
5	*L. noguchii*	Louisiana	Lousiana	LSU 1945
6	*L. noguchii*	Panama	Panama	CZ 214K
**7**	***L. biflexa***	**Semaranga**	**Patoc**	**Patoc 1**
**8**	***L. interrogans***	**Sejroe**	**Hardjo**	**Hardjo Prajitno**
**9**	***L. borgpetersenii***	**Tarassovi**	**Tarassovi**	**Perepelitsin**
**10**	***L. borgpetersenii***	**Ballum**	**Castellonis**	**Castellon**
**11**	***L. interrogans***	**Bataviae**	**Bataviae**	**Van Tienen**
12	*L. interrogans*	Sejroe	Wolfii	3705
13	*L. interrogans*	Pyrogenes	Pyrogenes	Salinem
**14**	***L. borgpetersenii***	**Ballum**	**Ballum**	**Mus 127**
**15**	***L. interrogans***	**Pomona**	**Pomona**	**Pomona**
16	*L.weilii*	Celledoni	Celledoni	Celledoni
17	*L. interrogans*	Autumnalis	Autumnalis	Akiyami A
**18**	***L. interrogans***	**Canicola**	**Canicola**	**H. Utrecht IV**
19	*L. kirschneri*	Cynopteri	Cynopteri	3522C
**20**	***L. kirschneri***	**Grippotyphosa**	**Grippotyphosa**	**Duyster**
21	*L. interrogans*	Hebdomadis	Hebdomadis	Hembomadis
**22**	***L. borgpetersenii***	**Mini**	**Mini**	**Sari**
23	*L. santarosai*	Shermani	Shermani	1342 K
24	*L. interrogans*	Australis	Bratislava	Jez Bratislava
**25**	***L. interrogans***	**Icterohaemorrhagiae**	**Copenhageni**	**L1 130**
26	*L. alexanderi*	Manhao	Manhao 3	L 60T
27	*L. alstoni*	Ranarum	Pingchang	80-412T
**28**	***L. kmetyi***	**Tarassovi**	**Malaysia**	**Bejo-Iso9**
29	*L. interrogans*	Pyrogenes	Manilae	L495
30	*L. mayottensis*	ND	ND	200901122
31	*L. santarosai*	Autumnalis	Canalzonae	AIM
32	*L. santarosai*	Grippotyphosa	Alice	JET
**33**	***L. sanjuanensis***	**ND**	**ND**	**LGVF01**

In bold: Final panel used for bovine serum samples.

The assays were conducted using dilution starting at 1:50 and 1:100 for human and domestic animals, respectively. A seropositive sample was considered when agglutination of leptospires was observed in ≥50% of the field of view at a titer of ≥1:100 for animals and ≥1:50 for humans, compared to negative controls. All seropositive samples were serial-diluted for titration to the endpoint of seropositivity. Additionally, the serogroup with the highest seropositivity titer, compared to the results of the other serogroups, was considered the probable source of exposure. Mixed seropositivity was defined as the presence of two or more serogroups with equal higher titers. All MAT procedures were conducted in accordance with the World Health Organization (WHO) guidelines. Briefly, live *Leptospira* reference strains were maintained under standard culture conditions and used as antigens in the assay. Quality control procedures, including the use of positive and negative control sera, were applied in accordance with WHO recommendations.

### Evaluation of Leptospira infection in canines and bovines

Urine samples from bovines via vulvar massage for females and preputial massage for males were collected. The area was cleaned with disposable wet wipes to induce spontaneous urination. The urine was initially stored in 15 mL tubes. For animals for which this method was not feasible, a swab of the external urinary meatus was taken using sterile plastic swabs and deposited in cryovials containing 0.9% saline solution. For canines, urine samples were collected through bladder catheterization. The prepuce or vulva of the animal was cleaned with sterile gauze pads soaked in a 0.5% chlorhexidine. Lidocaine spray as a topical anesthetic to minimize discomfort was applied. Catheterization was performed using 4**-**gauge Nelaton catheters that were lubricated with lidocaine gel.

All samples were stored in cryovials and placed in thermoses with liquid nitrogen for transport to Ciencias Veterinarias Centauro Laboratory at the Universidad de Antioquia, and finally stored at **-**80 °C. DNA extraction from urine samples were performed using a commercial Biospin Blood/Cell/Tissue Genomic DNA (Bioer) extraction and purification kit according to the manufacturer’s protocol. The final elution solution containing the extracted DNA was obtained and stored in 1.5**-**mL vials at −20 °C until processing by conventional and real-time PCR (RT-PCR).

The presence of inhibitors in all DNA samples was evaluated by PCR of the mitochondrial cytochrome B gene. All procedures were carried out according to Kocher et al. [15]. Additionally, RT-PCR was used to evaluate infection of *Leptospira* in the samples by 23S ribosomal gene specific to the pathogenic subclades pathogenic 1 (P1) and pathogenic 2 (P2) of *Leptospira*. DNA extracted from cultures of *L. santarosai* (P1) and *L. licerasiae* (P2) were used as positive controls. RT-PCR reactions were performed using a CFX96 Touch Real-Time PCR Detection System thermocycler (Bio-Rad) with TaqMan probes. The forward primer (PF) sequence was 5’-GACGAGGCTWAGWATGCG-3’, and the reverse primer (PR) sequence was 5’-ACCTAAACTTGAAAGMTATTTCTTT-3’. These primers were designed to amplify a 239 bp fragment. Two specific probes were also used for detection of P1 with the sequence 5’-CGGCTTATTGGTTGCCGTTG-3’, and P2 with the sequence 5’-ATGGTAATCCCCGTTGCGGA-3’. Real-time PCR was used as a qualitative assay to determine the presence or absence of *Leptospira* DNA. Samples were considered positive when amplification occurred with a cycle threshold (Ct) value below 32 cycles, as defined relative to the positive control.

### Assessment of Leptospira contamination in water and soil

Water samples were collected from water bodies located around each farm, mainly from lakes and drinking troughs inside animal pens. Sampling was performed near the shore at an approximate depth of 10 cm, collecting about 40 ml of water in 50 ml tubes. Samples were stored at 4 °C until processing. Soil samples were collected at the same locations as the water samples, ensuring the substrate was moist. These samples were also stored in 50 ml tubes and kept under the same transport and storage conditions as the water samples.

Specific kits were used for environmental samples depending on the type of matrix. BioFast Spin Water Genomic DNA Purification Kit for water samples and the BioFast Soil Genomic DNA Extraction Kit for soil samples were used. In both cases, 100**-**μL volumes of DNA were obtained and stored under the same conditions as the urine samples.

PCR of 16S rRNA gene was performed to evaluate the presence of inhibitors in all DNA samples, according to Weisburg et al. [16]. DNA extracted from *Ralstonia solanacearum* cultures was used as positive control. The same RT-PCR protocol of urine samples was used for the potential detection of leptospires in water and soil samples.

### Phylogenetic analysis

Several conventional PCRs were performed on the LipL31, LipL42, 16S rRNA [17–19], and 23S rRNA genes to identify the *Leptospira* species detected by RT-PCR. The fragments were sequenced using the Sanger method and edited using the SeqMan Pro tool in DNASTAR software. The edited sequences were analyzed using the Basic Local Alignment Search Tool (BLAST) program from the National Center for Biotechnology Information (NCBI). The consensus sequences were aligned with the reference sequences and other homologous *Leptospira* sequences using the MUSCLE algorithm in MEGA11. Phylogenetic reconstruction was performed using Bayesian phylogenetic analysis in MrBayes 3.2.7. The program ran for one million generations, displaying results every 500 generations. The K2P+G4 nucleotide substitution model was used and the best model was selected by the Bayesian Information Criterion (BIC) in the jModelTest 2.12 program. The resulting consensus tree was edited in FigTree v1.4.4.

### Definition of outcomes

The main outcomes were the seropositivity in bovines, humans, and canines, as well as the molecular detection of *Leptospira* in bovines, canines, water sources, and soil samples. The probable serogroup that represented the highest antibody titer was defined as the presumptive infecting or exposure serogroup. However, when two or more serogroups had the same higher titers, the sample was considered mixed serogroup seropositivity. Additionally, seropositivity outcome was categorized as a polytomous variable with both antibody titers and serogroups found.

### Statistical analysis

A descriptive analysis to characterize the bovine, human, and canine populations with respect to the study outcomes was performed. The Shapiro-Wilk test of quantitative variables to verify the assumption of normality was evaluated. The median and interquartile range were calculated for all quantitative variables without normality. For qualitative variables (dichotomous and polytomous), absolute and relative frequencies were calculated. Additionally, the proportion of seropositive and infection among humans, canines, and bovines was calculated by dividing the total number of seropositive individuals by the total number of individuals included in the study.

A Mixed Data Factor Analysis (MDFA) to explore the associations between seropositivity (including serogroups and titers) and *Leptospira* infection in bovines and productive and reproductive characteristics, as well as landscape metrics (quantitative and categorized variables) was made. The analysis also evaluated frequency of *Leptospira* seropositivity in canines and humans (serogroup of *Leptospira*). Variables included in the analysis were selected based on biological plausibility and the researchers’ criteria. The bovines age, bovines reproductive stage, *Leptospira* vaccination in bovines, use of biosecurity inputs, detection of leptospires in water and soil, total forest area, landscape proportion, number of patches, patch density; largest patch index, total edge, edge density, landscape shape index, topographic wetness index, pasture rotation, presence of canines in farm, presence of rodents in farm were variables included in the analysis.

To improve the interpretation of the MFDA, a stratified analysis was conducted according to criteria established in the scientific literature and quantitative variables categorized. The ranges reported as normal were considered according to the type of farm and the area of origin of the animals [20–22]. Five categories for the age in bovine variables were defined (≤ 1 year, > 1–4 years, > 4–7 years, > 7–10 years, and > 10 years). For landscape measurements, the results correspond to forest or dense vegetation for an estimation of forest fragmentation in each farm. These metrics showed highly anthropized landscapes with significant fragmentation. Therefore, landscape metrics were categorized based on the median value obtained from forest or dense vegetation classification, allowing for relative categorization adjusted to the study area’s particular conditions. To identify the associated variables, the categories of the closest variables were analyzed, considering the Euclidean distance by applying a nearest neighbor analysis in the MFDA. All statistical analyses were performed using R version 4.5.0.

## Results

### Epidemiological description of seropositivity/Infection by Leptospira in bovines

A total 494 bovines were sampled through the foot-and-mouth disease and brucellosis vaccination campaigns. The study included dry cows (44.9%; 222/494), growing heifer (18.6%; 92/494), recently calved cow (13%; 64/494), heifer calf (10.5%; 52/494), bull calf (8.1%; 40/494), replacement heifer (2.4%; 12/494), and bulls (0.8%; 4/494). Additionally, eight animals (1.6%; 8/494) were recorded with no available information. The median age in years was 4.13 (IQR: 1.11–6.84). Regarding breed composition, 68.8% (340/494) were Brahman; 17.0% (84/494) ¾ Angus × Brahman crosses, and 5.9% (29/494) were ¾ Brahman × Angus crosses. The median of age at first estrus was 26 months (IQR: 26.0–29.0), median age at first service was 27.5 months (IQR: 24.8–30.6), median age at first calving was 38.8 months (IQR: 35.6–41.9), median days open was 231 days (IQR: 108–295), the median calving interval was 430 days (IQR: 359–553) (Table 2).

**Table 2:** Characterization of cattle productive, reproductive, and management parameters.

Variables	Total N = 494 (%)	Seronegative N = 114 (%)	Seropositive N = 380 (%)	PCR Negative N = 474 (%)	PCR Positive N = 20 (%)
**Farm identification**					
Farm 1	71 (14.4)	15 (13.2)	56 (14.7)	71 (15.0)	0 (0)
Farm 2	25 (5.1)	3 (2.6)	22 (5.8)	25 (5.3)	0 (0)
Farm 3	53 (10.7)	8 (7.0)	45 (11.8)	53 (11.2)	0 (0)
Farm 4	102 (20.6)	17 (14.9)	85 (22.4)	88 (18.6)	14 (70.0)
Farm 5	100 (20.2)	28 (24.6)	72 (18.9)	99 (20.9)	1 (5.0)
Farm 6	54 (10.9)	11 (9.6)	43 (11.3)	52 (11.0)	2 (10.0)
Farm 7	89 (18.0)	32 (28.1)	57 (15.0)	86 (18.1)	3 (15.0)
**Age (in years)**					
Median [IQR]	4.13 [1.11, 6.84]	1.20 [0.734, 6.52]	4.29 [1.45, 6.91]	4.26 [1.12, 6.93]	1.33 [1.11, 1.59]
**Reproductive status**					
Heifer calf	52 (10.5)	22 (19.3)	30 (7.9)	51 (10.8)	1 (5.0)
Bull calf	40 (8.1)	17 (14.9)	23 (6.1)	39 (8.2)	1 (5.0)
Growing heifer	92 (18.6)	19 (16.7)	73 (19.2)	77 (16.2)	15 (75.0)
Replacement heifer	12 (2.4)	2 (1.8)	10 (2.6)	12 (2.5)	0 (0)
Recently calved cow	64 (13.0)	11 (9.6)	53 (13.9)	63 (13.3)	1 (5.0)
Dry cow	222 (44.9)	41 (36.0)	181 (47.6)	221 (46.6)	1 (5.0)
Bull	4 (0.8)	0 (0)	4 (1.1)	4 (0.8)	0 (0)
No data	8 (1.6)	2 (1.8)	6 (1.6)	7 (1.5)	1 (5.0)
**Number of calvings**					
≤ 4 calvings	208 (42.1)	39 (34.2)	169 (44.5)	206 (43.5)	2 (10.0)
≥ 5 calvings	78 (15.8)	13 (11.4)	65 (17.1)	78 (16.5)	0 (0)
Not applicable	200 (40.5)	60 (52.6)	140 (36.8)	183 (38.6)	17 (85.0)
No data	8 (1.6)	2 (1.8)	6 (1.6)	7 (1.5)	1 (5.0)
**Age at first breeding (in months)**					
Median [IQR]	26.0 [23.0, 29.0]	25.0 [23.0, 29.0]	26.0 [23.0, 29.0]	26.0 [23.0, 29.0]	24.5 [22.3, 26.8]
**Age at first service (in months)**					
Median [IQR]	27.5 [24.8, 30.6]	27.4 [25.1, 30.9]	27.5 [24.6, 30.6]	27.5 [24.9, 30.7]	25.3 [23.2, 27.4]
**Age at first calving (in months)**					
Median [IQR]	38.8 [35.6, 41.9]	38.6 [35.2, 41.3]	38.8 [35.6, 42.2]	38.8 [35.6, 41.9]	36.0 [34.5, 37.4]
**Services per conception**					
1	93 (18.8)	21 (18.4)	72 (18.9)	93 (19.6)	0 (0)
2	153 (31.0)	26 (22.8)	127 (33.4)	152 (32.1)	1 (5.0)
> 2	31 (6.3)	4 (3.5)	27 (7.1)	31 (6.5)	0 (0)
Not applicable	188 (38.1)	58 (50.9)	130 (34.2)	171 (36.1)	17 (85.0)
No data	29 (5.9)	5 (4.4)	24 (6.3)	27 (5.7)	2 (10.0)
**Days open**					
Median [IQR]	231 [108, 295]	247 [123, 300]	225 [106, 294]	231 [109, 295]	130 [80.0, 180]
**Calving interval (in days)**					
Median [IQR]	430 [359, 553]	472 [387, 592]	426 [355, 543]	426 [359, 553]	554 [516, 591]
**Weaning weight (kg)**					
Median [IQR]	190 [170, 206]	190 [175, 210]	190 [170, 204]	190 [170, 207]	180 [166, 190]
**Body condition score (1 –5)**					
< 3	63 (12.8)	13 (11.4)	50 (13.2)	63 (13.3)	0 (0)
3 to 3.5	192 (38.9)	33 (28.9)	159 (41.8)	190 (40.1)	2 (10.0)
> 3.5	28 (5.7)	5 (4.4)	23 (6.1)	28 (5.9)	0 (0)
No data	211 (42.7)	63 (55.3)	148 (38.9)	193 (40.7)	18 (90.0)
**Average daily gain (g)**					
Median [IQR]	250 [165, 548]	301 [163, 565]	246 [166, 520]	243 [163, 521]	630 [546, 669]
**Breed**					
3/4 Angus	84 (17.0)	25 (21.9)	59 (15.5)	81 (17.1)	3 (15.0)
3/4 Brahman	29 (5.9)	11 (9.6)	18 (4.7)	28 (5.9)	1 (5.0)
3/4 Gyr	5 (1.0)	1 (0.9)	4 (1.1)	5 (1.1)	0 (0)
Brahman	340 (68.8)	70 (61.4)	270 (71.1)	325 (68.6)	15 (75.0)
F1 Angus	15 (3.0)	3 (2.6)	12 (3.2)	15 (3.2)	0 (0)
F1 Brahman	3 (0.6)	1 (0.9)	2 (0.5)	3 (0.6)	0 (0)
F1 Simmental	1 (0.2)	1 (0.9)	0 (0%)	1 (0.2%)	0 (0)
1/4 Angus	1 (0.2)	0 (0)	1 (0.3)	1 (0.2)	0 (0)
3/4 Guzerat	2 (0.4)	0 (0)	2 (0.5)	2 (0.4)	0 (0)
Angus	3 (0.6)	0 (0)	3 (0.8)	3 (0.6)	0 (0)
F1 Guzerat	1 (0.2)	0 (0)	1 (0.3)	1 (0.2)	0 (0)
F1 Holstein	2 (0.4)	0 (0)	2 (0.5)	2 (0.4)	0 (0)
No data	8 (1.6)	2 (1.8)	6 (1.6)	7 (1.5)	1 (5.0)
**Reproductive disorders**					
Abortion	10 (2.0)	1 (0.9)	9 (2.4)	10 (2.1)	0 (0)
Stillbirth	8 (1.6)	1 (0.9)	7 (1.8)	8 (1.7)	0 (0)
Resorption	2 (0.4)	1 (0.9)	1 (0.3)	2 (0.4)	0 (0)
**Type of grass**					
*Dichanthium aristatum benth*	149 (30.2)	38 (33.3)	111 (29.2)	146 (30.8)	3 (15.0)
*Dichantium aristatum benth +* *Urochloa arrecta +* Botswana	25 (5.1)	3 (2.6)	22 (5.8)	25 (5.3)	0 (0)
*Dichantium aristatum benth* + *Bothriochloa pertusa + Panicum maximum* cv. Mombasa)	100 (20.2)	16 (14.0)	84 (22.1)	87 (18.4)	13 (65.0)
*Dichantium aristatum benth +* *Bothriochloa pertusa +* *Panicum maximum* cv. Mombasa + *Urochloa brizantha + Urochloa decumbens cv basilisk +* *Urochloa arrecta*	71 (14.4)	15 (13.2)	56 (14.7)	71 (15.0)	0 (0)
*Bothriochloa pertusa + Dichantium aristatum benth + Dichantium annulatum*	52 (10.5)	8 (7.0)	44 (11.6)	52 (11.0)	0 (0)
*Ischaemum ciliare +* *Dichantium annulatum*	89 (18.0)	32 (28.1)	57 (15.0)	86 (18.1)	3 (15.0)
**Proper feed storage**	441 (89.3)	106 (93.0)	335 (88.2)	421 (88.8)	20 (100)
***Leptospira* vaccination performed**	71 (14.4)	15 (13.2)	56 (14.7)	71 (15.0)	0 (0)
**Implementation of biosecurity measures**	363 (73.5)	86 (75.4)	277 (72.9)	346 (73.0)	17 (85.0)
**Paddock rotation schedules (in days)**					
3	100 (20.2)	16 (14.0)	84 (22.1)	87 (18.4)	13 (65.0)
5	193 (39.1)	51 (44.7)	142 (37.4)	188 (39.7)	5 (25.0)
6	25 (5.1)	3 (2.6)	22 (5.8)	25 (5.3)	0 (0)
7	97 (19.6)	27 (23.7)	70 (18.4)	96 (20.3)	1 (5.0)
8	71 (14.4)	15 (13.2)	56 (14.7)	71 (15.0)	0 (0)
**Presence of other animals in paddocks**					
Canines	174 (35.2)	41 (36.0)	133 (35.0)	171 (36.1)	3 (15.0)
Swine	97 (19.6)	27 (23.7)	70 (18.4)	96 (20.3)	1 (5.0)
Poultry	97 (19.6)	27 (23.7)	70 (18.4)	96 (20.3)	1 (5.0)
Turkeys	97 (19.6)	27 (23.7)	70 (18.4)	96 (20.3)	1 (5.0)
Horses	338 (68.4)	86 (75.4)	252 (66.3)	319 (67.3)	19 (95.0)
Donkeys	149 (30.2)	38 (33.3)	111 (29.2)	146 (30.8)	3 (15.0)
Mules	149 (30.2)	38 (33.3)	111 (29.2)	146 (30.8)	3 (15.0)
Rodents	97 (19.6)	27 (23.7)	70 (18.4)	96 (20.3)	1 (5.0)

A proportion of seropositivity against *Leptospira* 76.9% (380/494) was calculated. The median age of the seropositive animals was 4.29 years (IQR: 1.45–6.91). Dry cows exhibited the highest seropositivity frequency (47.6%; 181/380), growing heifers of 19.2% (73/380) and recently calved cows was 13.9% (53/380). Among seropositive animals, 22.4% (85/380) came from farm 4, 18.8% (72/380) from farm 5, 15% (57/380) from farm 7, 14.7% (56/380) from farm 1, 11.8% (45/380) from farm 3, 11.3% (43/380) from farm 6, and 5.8% (22/380) came from farm 2 (Table 2).

Among all case seropositives, the most frequent serogroup was Tarassovi (31.3%; 119/380), followed by Mini serogroup (30.2%;115/380) and mixed seropositivity (17.9%; 68/380). In addition, the antibody titers found ranged from 1:100–1:3200. The most frequent titer was 1:400 (28.4%; 108/380), followed by 1:200 (24.2%; 92/380) and 1:800 (21.05%; 80/380).

Regarding the infection by *Leptospira*, the proportion of *Leptospira* infection 4.04% (20/494) was calculated. Of these, 70% (14/20) came from farm 4, 15% (3/20) from farm 7, 10% (2/20) from farm 6, and 5% (1/20) came from farm 5 (Table 2). The median age of infected animals was 1.33 years (IQR: 1.11–1.59). Growing heifers represented the reproductive group with the most frequency of *Leptospira* infection (75%; 15/20). Additionally, Brahman breed had the most frequency of positivity (75%;15/20) (Table 2). The sequences of 16S ribosomal DNA samples had identity percentages between 97% to 100% with *Leptospira borgpetersenii* and alignment coverage ranging from 98% to 100%.

### Epidemiological description of seropositivity against Leptospira in humans

A total of 73 individuals were enrolled in this study, with a sampling coverage of 67.59% (73/108). Among these individuals, 56.2% (41/73) and 43.8% (32/73) were male and female, respectively. The median age in year was 33 (IQR: 22.0–43.0), and the median time living in the area was 4.25 years (IQR: 1.00–8.00). A proportion of seropositivity of *Leptospira* of 4.1% (3/73) was calculated. The serogroups Autumnalis, Icterohaemorrhagiae, and Sejroe (all with titers of 1:50) were identified. All of the seropositive individuals lived or worked on farms 3, 4, and 7. Two seropositive individuals were male, and the median age in years was 29 (IQR: 21.5–40.5). Among seropositive individuals, animal caretaker, student, and domestic work were occupations reported. Other characteristics of epidemiological importance for *Leptospira* exposure of/transmission such as attitude and practices are shown in Table 3.

**Table 3:** Characterization of the study participants and their households.

Variables	Total N = 73 (%)	Seronegative N = 70 (%)	Seropositive N = 3 (%)
**Farm identification**			
Farm 1	14 (19.2)	14 (20.0)	0 (0)
Farm 2	7 (9.6)	7 (10.0)	0 (0)
Farm 3	9 (12.3)	8 (11.4)	1 (33.3)
Farm 4	11 (15.1)	10 (14.3)	1 (33.3)
Farm 5	12 (16.4)	12 (17.1)	0 (0)
Farm 6	7 (9.6)	7 (10.0)	0 (0)
Farm 7	13 (17.8)	12 (17.1)	1 (33.3)
**Gender**			
Male	41 (56.2)	39 (55.7)	2 (66.7)
Female	32 (43.8)	31 (44.3)	1 (33.3)
**Age (in years)**			
Median [IQR]	33.0 [22.0, 43.0]	33.5 [22.0, 43.0]	29.0 [21.5, 40.5]
**Ethnicity**			
Indigenous	1 (1.4)	1 (1.4)	0 (0)
Afro-descendant	5 (6.8)	5 (7.1)	0 (0)
None	58 (79.5)	56 (80.0)	2 (66.7)
Not sure	9 (12.3)	8 (11.4)	1 (33.3)
**Time of living in the area (in years)**			
Median [IQR]	4.25 [1.00, 8.00]	5.00 [1.16, 8.00]	1.00 [1.00, 2.50]
**Level of education**			
No formal education	3 (4.1)	2 (2.9)	1 (33.3)
Primary education in progress	7 (9.6)	7 (10.0)	0 (0)
Completed primary education	9 (12.3)	9 (12.9)	0 (0)
Incomplete primary education	7 (9.6)	7 (10.0)	0 (0)
Secondary education in progress	6 (8.2)	5 (7.1)	1 (33.3)
Completed secondary education	13 (17.8)	13 (18.6)	0 (0)
Incomplete secondary education	13 (17.8)	12 (17.1)	1 (33.3)
Technical education in progress	2 (2.7)	2 (2.9)	0 (0)
Technology education in progress	1 (1.4)	1 (1.4)	0 (0)
Completed technical education	8 (11.0)	8 (11.4)	0 (0)
Completed undergraduate education	3 (4.1)	3 (4.3)	0 (0)
Completed graduate education	1 (1.4)	1 (1.4)	0 (0)
**Professional career**			
No professional career	58 (79.5)	55 (78.6)	3 (100)
Technical, technological or professional	15 (20.5)	15 (21.4)	0 (0)
**Occupation**			
Farm manager	7 (9.6)	7 (10.0)	0 (0)
Unemployed	3 (4.1)	3 (4.3)	0 (0)
Animal caretaker	20 (27.4)	19 (27.1)	1 (33.3)
Student	16 (21.9)	15 (21.4)	1 (33.3)
Domestic duties	21 (28.8)	20 (28.6)	1 (33.3)
Miscellaneous tasks	4 (5.5)	4 (5.7)	0 (0)
Veterinarian	2 (2.7)	2 (2.9)	0 (0)
**Workplace**			
Within facilities	40 (54.8)	38 (54,2)	2 (66,7)
Outdoors	18 (24.7)	17 (24,3)	1 (33,3)
Both	12 (16.4)	12 (17.1)	0 (0)
Not applicable (unemployed)	3 (4.1)	3 (4.3)	0 (0)
**Contact with animal urine or feces**	40 (54.8)	38 (54.3)	2 (66.7)
**Contact with placentas and newborn animals**	30 (41.1)	29 (41.4)	1 (33.3)
**Contact with animal food or water**	37 (50.7)	36 (51.4)	1 (33.3)
**Contact with streams, lakes and creeks**	36 (49.3)	34 (48.6)	2 (66.7)
**Contact with sewage water**	13 (17.8)	12 (17.1)	1 (33.3)
**Use of gloves**	18 (24.7)	17 (24.3)	1 (33.3)
**Hand washing**	46 (63.0)	44 (62.9)	2 (66.7)
**To get to the workplace, pass through**			
Forests	12 (16.4)	11 (15.7)	1 (33.3)
Paddocks	27 (37.0)	25 (35.7)	2 (66.7)
Creeks	21 (28.8)	19 (27.1)	2 (66.7)
**Walking barefoot**	25 (34.2)	25 (35.7)	0 (0)
**Bathing in natural water sources**	21 (28.8)	20 (28.6)	1 (33.3)
**Drinking water from natural sources**	59 (80.8)	57 (81.4)	2 (66.7)
**Fever in the last year**	34 (46.6)	33 (47.1)	1 (33.3)
**Reported knowledge of rat fever**	26 (35.6)	25 (35.7)	1 (33.3)
**Reported perceiving rats as dangerous**	57 (78.1)	54 (77.1)	3 (100)
**Housing location**			
Rural	63 (86.3)	60 (85.7)	3 (100)
Urban	3 (4.1)	3 (4.3)	0 (0)
No data	7 (9.6)	7 (10)	0 (0)
**Spatial arrangement of the housing**			
Highly dispersed	55 (75.3)	52 (74.3)	3 (100)
Dispersed	9 (12.3)	9 (12.9)	0 (0)
Concentrated	1 (1.4)	1 (1.4)	0 (0)
Highly concentrated	1 (1.4)	1 (1.4)	0 (0)
**Roof material**			
Vegetal material roof	38 (52.1)	36 (51.4)	2 (66.7)
Zinc sheet roof	25 (34.2)	23 (32.9)	2 (66.7)
Clay tile roof	11 (15.1)	10 (14.3)	1 (33.3)
Wooden roof	17 (23.3)	17 (24.3)	0 (0)
**Floor material**			
Cement floor	57 (78.1)	54 (77.1)	3 (100)
Dirt floor	16 (21.9)	14 (20.0)	2 (66.7)
Tile floor	8 (11.0)	7 (10.0)	1 (33.3)
**Wall material**			
Block Wall	19 (26.0)	17 (24.3)	2 (66.7)
Wooden Wall	44 (60.3)	43 (61.4)	1 (33.3)
Cement Wall	3 (4,1)	3 (4,3)	0 (0)
**Public utilities available in the dwelling**			
Potable water	21 (28.8)	20 (28.6)	1 (33.3)
Sewage system	1 (1.4)	1 (1.4)	0 (0)
Septic tank	58 (79.5)	55 (78.6)	3 (100)
Latrine	5 (6.8)	5 (7.1)	0 (0)
**Source of water for household use**			
Urban water supply system	9 (12.3)	9 (12.9)	0 (0)
Rural water supply system	42 (57.5)	39 (55.7)	3 (100)
Rainwater	36 (49.3)	35 (50.0)	1 (33.3)
**Household water storage**			
In a pump well	26 (35.6)	24 (34.3)	2 (66.7)
In a well without a pump	6 (8.2)	6 (8.6)	0 (0)
In an elevated tank	41 (56.2)	39 (55.7)	2 (66.7)
In an underground tank	3 (4.1)	3 (4.3)	0 (0)
In buckets or drums	5 (6.8)	5 (7.1)	0 (0)
No water storage	4 (5.5)	4 (5.7)	0 (0)
**Treatment of drinking water is performed before consumption**	21 (28.8)	21 (30.0)	0 (0)
**Type of treatment**			
Water boiling	14 (19.2)	14 (20.0)	0 (0)
Chlorination	7 (9.6)	7 (10.0)	0 (0)
Water filtration	2 (2.7)	2 (2.9)	0 (0)
**Waste disposal**			
Waste collection truck	10 (13.7)	10 (14.3)	0 (0)
Waste burning	45 (61.6)	43 (61.4)	2 (66.7)
Waste burial	17 (23.3)	16 (22.9)	1 (33.3)
Used as animal feed	12 (16.4)	11 (15.7)	1 (33.3)
Disposal into the river	4 (5.5)	4 (5.7)	0 (0)
Composting	15 (20.5)	14 (20.0)	1 (33.3)
**Natural water sources near the household**			
Lake	6 (8.2)	6 (8.6)	0 (0)
River	19 (26.0)	17 (24.3)	2 (66.7)
Stream	33 (45.2)	32 (45.7)	1 (33.3)
Sewage pipe	5 (6.8)	5 (7.1)	0 (0)
Drainage ditch	8 (11.0)	8 (11.4)	0 (0)
**Rainwater storage containers**	33 (45.2)	31 (44.3)	2 (66.7)
Tank	15 (20.5)	15 (21.4)	0 (0)
Bucket or drum	7 (9.6)	6 (8.6)	1 (33.3)
Hole or cavity	9 (12.3)	9 (12.9)	0 (0)
Well	9 (12.3)	8 (11.4)	1 (33.3)
**Presence of flood barriers**	53 (72.6)	51 (72.9)	2 (66.7)
**Barriers types**			
Raised floors	34 (46.6)	33 (47.1)	1 (33.3)
Exterior walls	19 (26.0)	19 (27.1)	0 (0)
Drains and ditches	48 (65.8)	46 (65.7)	2 (66.7)
**Green area in the peridomicile**			
Shrub	37 (50.7)	36 (51.4)	1 (33.3)
Tree	36 (49.3)	35 (50.0)	1 (33.3)
Grass	25 (34.2)	24 (34.3)	1 (33.3)
Plantain crop	44 (60.3)	42 (60.0)	2 (66.7)
Coconut crop	33 (45.2)	32 (45.7)	1 (33.3)
Corn crop	11 (15.1)	9 (12.9)	2 (66.7)
Rice crop	4 (5.5)	3 (4.3)	1 (33.3)
Yam crop	1 (1.4)	1 (1.4)	0 (0)
Cassava crop	24 (32.9)	22 (31.4)	2 (66.7)
**Animals in the peridomicile**			
Canines	47 (64.4)	44 (62.9)	3 (100)
Felines	35 (47.9)	33 (47.1)	2 (66.7)
Poultry	58 (79.5)	55 (78.6)	3 (100)
Swine	42 (57.5)	39 (55.7)	3 (100)
Turkeys	10 (13.7)	9 (12.9)	1 (33.3)
Horses	27 (37.0)	25 (35.7)	2 (66.7)
Donkeys	2 (2.7)	2 (2.9)	0 (0)
Mules	7 (9.6)	6 (8.6)	1 (33.3)
Rodents	47 (64.4)	45 (64.3)	2 (66.7)
Opossums	48 (65.8)	46 (65.7)	2 (66.7)
Wildlife	37 (50.7)	34 (48.6)	3 (100)
**Protective measures to prevent rat infestation**			
Filling puddles with soil	16 (21.9)	15 (21.4)	1 (33.3)
Cleaning campaigns	20 (27.4)	19 (27.1)	1 (33.3)
Self-protection	22 (30.1)	21 (30.0)	1 (33.3)
Rodenticide	40 (54.8)	38 (54.3)	2 (66.7)
Ditch cleaning	48 (65.8)	45 (64.3)	3 (100)
**Agriculture in forests**	11 (15.1)	10 (14.3)	1 (33.3)
**Hunting of wildlife**	5 (6.8)	5 (7.1)	0 (0)
Hunting of wildlife for food	5 (6.8)	5 (7.1)	0 (0)
Hunting of wildlife for defense	3 (4.1)	3 (4.3)	0 (0)

### Epidemiological description of seropositivity/Infection by Leptospira in canines

A total of 15 canines were sampled, resulting in a sampling coverage of 71.42% (15/21). Regarding canine breed, 80% (12/15) were mixed breed, 13.3% (2/15) German Shepherds, and 6.7% (1/15) were German Shepherd mixes. Also, 66% (10/15) were male, while 33.3% (5/15) were female. Three animals were less than one year old (20%), eight were between one and two years old (53.3%), and four were over two years old (26.7%). 87.6% (13/15) of the canines had remained on the farms since birth. Only one of the canines had leptospirosis vaccination at the time of sample collection, and 60% (9/15) had internal deworming (Table 4).

**Table 4:** Characteristics of the canine population enrolled in the study.

Variables	Total N = 15 (%)	Seronegative N = 10 (%)	Seropositive N = 5 (%)
**Breed**			
Mixed-breed	12 (80.0)	7 (70.0)	5 (100)
German Shepherd mix	1 (6.7)	1 (10.0)	0 (0)
German Shepherd	2 (13.3)	2 (20.0)	0 (0)
**Age (in years)**			
< 1	3 (20.0)	2 (20.0)	1 (20.0)
1–2	8 (53.3)	5 (50.0)	3 (60.0)
> 2	4 (26.7)	3 (30.0)	1 (20.0)
**Body condition score (1 –9)**			
4	9 (60.0)	6 (60.0)	3 (60.0)
5	3 (20.0)	3 (30.0)	0 (0)
6	3 (20.0)	1 (10.0)	2 (40.0)
**Born on the farm**			
No	13 (86.7)	10 (100)	3 (60.0)
Yes	2 (13.3)	0 (0)	2 (40.0)
***Leptospira* vaccination performed**	1 (6.7)	0 (0)	1 (20.0)
**Internal deworming performed**	9 (60.0)	5 (50.0)	4 (80.0)
**Potable water consumption**	1 (6.7)	1 (10.0)	0 (0)
**Used as companion animal**	14 (93.3)	10 (100)	4 (80.0)
**Mainly kept outdoors**	6 (40.0)	3 (30.0)	3 (60.0)
**Contact with cattle**	5 (33.3)	2 (20.0)	3 (60.0)
**Contact with rodents**	6 (40.0)	3 (30.0)	3 (60.0)

The proportion of seropositivity of *Leptospira* was 33.3% (5/15). Two of these seropositive canines lived in farm 3, and three lived in farms 1, 4, and 6. All seropositive canines were mixed breeds and 60% (3/5) were one and two years old. Finally, only one had the *Leptospira* vaccination protocol, and four canines had current internal deworming (Table 4).

According to the serogroups identified, three canines were exposed to Canicola (titres of 1:3200), Autumnalis (titres of 1:200), and Icterohaemorrhagiae (titres of 1:400, vaccinated individual), and two were confirmed to be mixed seropositive. No infection was identified in any urine samples from these animals.

### Molecular detection of Leptospires in water and soil

**Fig 2:**
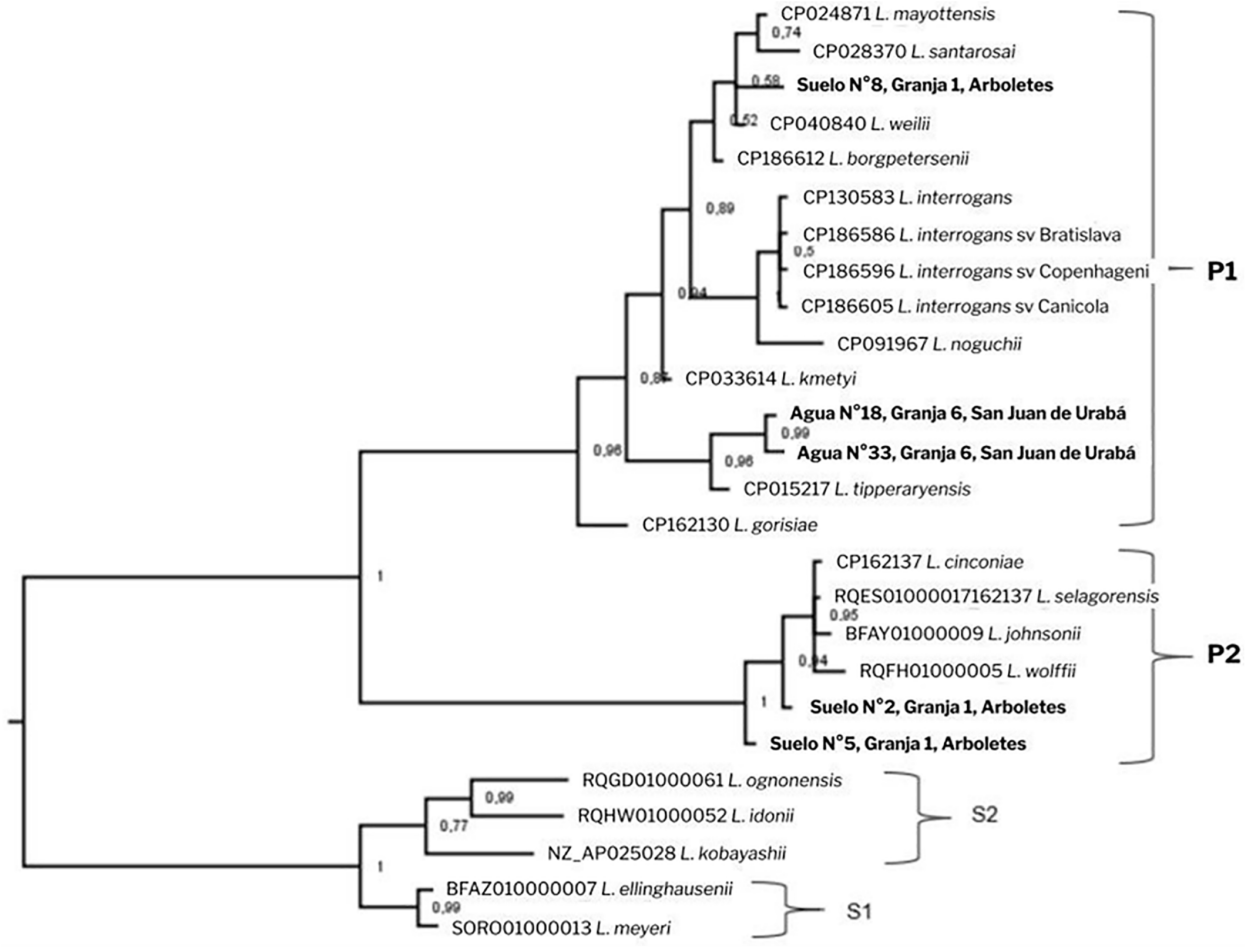
Bayesian phylogenetic tree of the 23S ribosomal gene of *Leptospira* detected in water and soil. The sequences obtained in this study are highlighted in red. The numbers near the nodes represent probability values. The substitution model used was K2P+G4. The analysis was performed using MrBayes v3.2.7.

A proportion of *Leptospira* contamination in water sources of 7.5% (3/40) was calculated. One contaminated source was found on farm 5, and two were found on farm 6. Two 23S rRNA sequences showed 97.2%**-**97.8% identity with *Leptospira tipperaryensis*, a species belonging to pathogenic subclade 1 (GenBank: PX957500, PX95750). Additionally, these sequences had 100% alignment coverage. However, one water sample from Farm 5 showed low percentages of identity and coverage, which prevented accurate identification of the *Leptospira* species (Fig 2).

Regarding the soil samples, a proportion of Leptospira contamination of 7.6% (3/39) was found. In this sense, all samples were collected from Farm 1. Two 23S rRNA sequences showed 97.5% and 98.7% identity with *Leptospira cinconiae*, a species belonging to pathogenic subclade 2 (100% alignment coverage) (GenBank: PX957502, PX957503). The third sample was more closely related to *Leptospira weilii* from pathogenic subclade 1, with 98.6% identity and 99% coverage (GenBank: PX957504) (Fig 2).

### Landscape metrics for land use and land cover classes

All farms exhibited significant alterations in landscape metrics and land cover, suggesting highly fragmented environments potentially resulting from historical livestock farming and extensive agricultural practices. In this regard, farm 1 recorded total forest or dense vegetation of 20.19% of the total farm area, with low representativeness in the landscape. This forest exhibited high fragmentation, characterized by a large number of patches (5,300), high patch density (3,979.09 patches/100 ha), and complex spatial configuration. Most of this farm’s land was used for pastures, including *Dichantium aristatum, Bothriochloa pertusa, Panicum maximum* cv. Mombasa, *Urochloa brizantha, Urochloa decumbens* cv. Basilisk, and *Urochloa arrecta*. Additionally, there was a total of 105 ha of pasture and 26.9 ha of forest or dense vegetation, including various types of forests, which were often tertiary or quaternary (S1 Fig and S2 Table).

**Fig 3:**
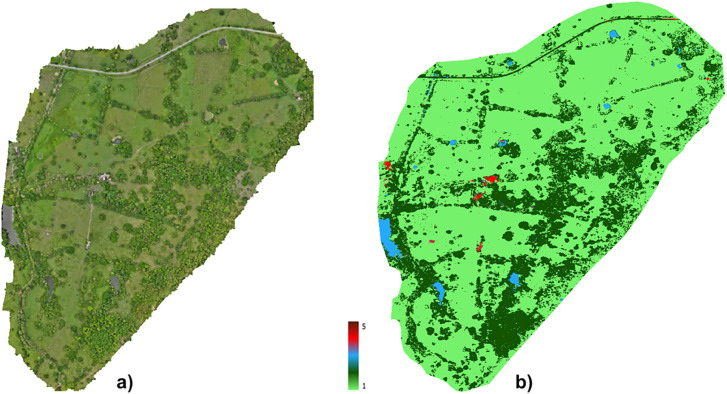
Orthomosaic and land classification of the Farm 2. **A)** Orthomosaic of the Farm 2. **(B)** Land classification of the Farm 2, showing: pastures (light green), dense vegetation (dark green), water bodies (blue) and built-up areas (red) (automatic classification using Python version 3.9). The orthomosaic was created by manually delineating the farm boundaries using an unmanned aerial vehicle to collect the data, and the analysis was performed using Agisoft Metashape 1.8 software. Orthomosaic is provided in KMZ and can be visualized using Google Earth Pro.

Furthermore, on Farm 2, a total area covered by forest or dense vegetation of 28.28% of the total area was found, with an absolute representation of the landscape of 50.63 ha. The forest exhibited a high degree of fragmentation (10,701 patches), a high patch density (5,977.47 patches/100 ha), and a complex spatial configuration. Most of the area of this farm was occupied by pastures (Fig 3 and Table 5).

**Table 5:** Description of the landscape metrics for Farm 2.

Land use cover class	Total area (ha)	Landscape proportion (%)	Number of patches	Patch density (patches per 100 ha)	Largest patch index (%)	Total edge (m)	Edge density (m/ha)	Landscape shape index
Pasture or forage	125.79	70.26	11470	6407.02	62.92	264451.01	1477.19	60.25
Forest or dense vegetation	50.63	28.28	10701	5977.47	7.73	273469.79	1527.57	95.77
Water bodies	2.03	1.13	2279	1273.02	0.51	11979.26	66.91	21.14
Built-up areas	0.55	0.31	341	190.47	0.06	5212.71	29.11	17.34
Crop cultivation	0.0004	0.0002	1	0.55	0.0002	10.20	0.05	1.18

In contrast, 11.34% of the total area of Farm 3 was covered by forest or dense vegetation, representing 30.09 ha of the landscape. The forest exhibited a high level of fragmentation, with 15,705 patches and a density of 5,921.56 patches/100 ha, as well as a complex spatial configuration. It also exhibited a substantial length of forest edges (277,036.15 m) and a high edge density (1,044.56 m/ha). Most of this farm’s land was used as pasture, specifically for *Bothriochloa pertusa*, *Dichantium aristatum Benth*. and *Dichantium annulatum*, covering an area of 233.94 ha (S2 Fig and S3 Table).

In addition, Farm 4 had reduced total vegetation cover and average representation in the landscape. The forest was highly fragmented and characterized by a large number of patches (5,566), a high patch density (4,121.50 patches/100 ha) and long edge lengths (135,918 m). It had a complex spatial configuration and a total area of dense vegetation of 44.22 ha. In addition, farm 4 had reduced total vegetation cover and average representation in the landscape. The majority of this Farm was used as pasture (61.23 ha), specifically for *Dichantium aristatum benth, Bothriochloa pertusa,* and *Panicum maximum* cv. Mombasa (S3 Fig and S4 Table).

Similarly, Farm 5 featured a small area of forest or dense vegetation that had low representativeness in the landscape. The forest was highly fragmented, consisting of a large number of patches (20,622), a very high patch density (9,125.59 patches/100 ha) and a high edge length (396,665.09 m), as well as a complex spatial configuration. It also had a high length and density of forest edges. Forest or dense vegetation covered 47.16 ha of the total farm area. Most of the farm was occupied by pastures (176.15 ha), specifically *Dichantium aristatum Benth* (S4 Fig and S5 Table).

A total area of forest or dense vegetation was found on farm 6, representing 36.98% of the total farm landscape. The forest exhibited a high level of fragmentation, featuring 9,598 patches and a total edge length of 222,010.39 m. Also, a very high patch density of 8,273.48 patches/100 ha and a highly complex spatial configuration was found. Most of the area of this farm was occupied by pastures of *Dichantium aristatum benth*, representing 61.98% of the total landscape (S5 Fig and S6 Table).

Finally, an area of forest or dense vegetation representing 24.83% of the landscape was found on Farm 7. The forest was highly fragmented, consisting of 392 patches with a patch density of 355.07 patches/100 ha. It also had a total edge length of 47,116.39 m and a highly complex spatial configuration. Most of the farm area was occupied by pastures, specifically, *Ischaemum ciliare* and *Dichantium annulatum*, accounting for 74.42% of the total area (S6 Fig and S7 Table).

### Topographic Wetness Index (TWI) based on the digital elevation model (DEM)

In general, the farms had large areas susceptible to flooding, which could favor the spread of leptospires in soil and bodies of water available for consumption by humans and animals. In this regard, Farm 1 had a total floodable area of 26.37 hectares, accounting for 19.5% of the total area of 134.91 hectares (S8 Fig).

**Fig 4:**
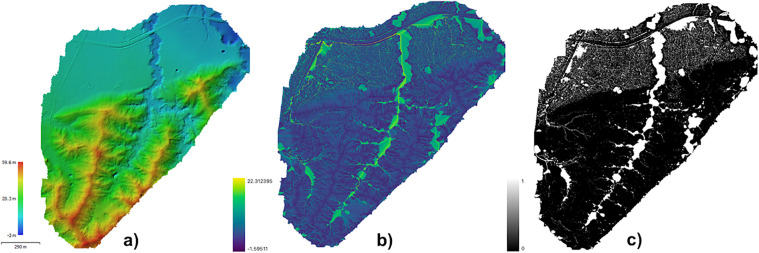
Digital elevation model and flooded area of the Farm 2. **(A)** Digital elevation model of the Farm 2. **(B)** Flooded area of Farm 2. **(C)** Proportion of floodable area of the Farm 2. The base map was developed through the manual delineation of farm boundaries by the research team. The map was created in QGIS software. Digital elevation model is provided in KMZ and can be visualized using Google Earth Pro.

A total floodable area of 27.98 hectares was determined for Farm 2, representing 15.20% of the total area (183.41 hectares) (Fig 4). Similarly, in Farm 3, the total floodable area was determined to be 38.16 hectares, representing 13.9% (areas with TWI > 10) of the total farm area (274.19 hectares) (S9 Fig). On Farm 4, the total floodable area was 15.85 hectares, representing 6.3% (areas with TWI > 10) of the 252.80-hectare farm (S10 Fig).

A total floodable area of 43.36 hectares was determined on Farm 5, representing 18.9% of the total area (229.86 hectares) (S11 Fig). On farm 6, the total floodable area was found to be 10.06 hectares, accounting for 8.7% (areas with TWI > 10) of the property’s total area (115.40 hectares) (S12 Fig). Finally, on Farm 7, a total floodable area of 15.69 hectares was determined, accounting for 17.8% of the total area (88.21 hectares) (S13 Fig).

### Mixed Data Factor Analysis (MFDA)

**Fig 5:**
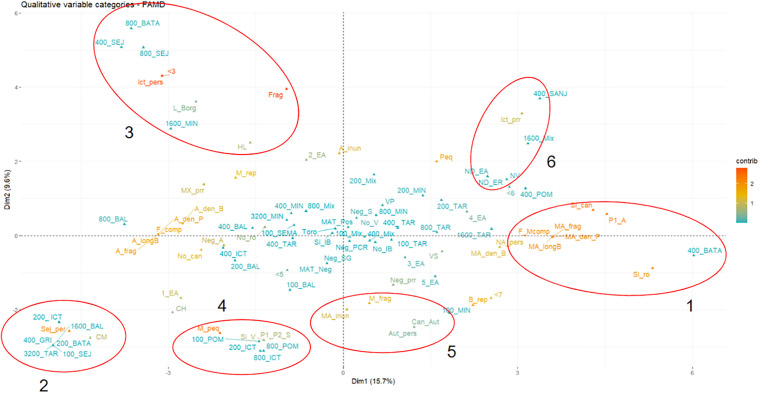
Contribution of qualitative variables and associations based on nearest neighbor analysis in the MDFA. MA_longB: very high edge length, A_longB: high edge length, MA_den_B: very high edge density, A_den_B: high edge density, MA_frag: very high fragmentation, A_frag: high fragmentation, MA_den_P: very high patch density, A_den_P: high patch density, F_Mcomp: very complex landscape shape, F_comp: complex landscape shape, M_rep: medium representation of dense vegetation area in the landscape, B_rep: low representation of dense vegetation area in the landscape, M_peq: very small dense vegetation area, Peq: small dense vegetation area, Frag: largest fragmented patch, M_frag: largest highly fragmented patch, MA_inun: very highly floodable landscape, A_inun: highly floodable landscape, < 7: paddock rotation every 7 days, < 6: paddock rotation every 6 days, < 5: paddock rotation every 5 days, < 3: paddock rotation every 3 days, P1_A: contamination by *Leptospira* of subclade P1 in water sources, Neg_A: water with no detection of pathogenic *Leptospira* contamination, P1_P2_S: contamination by *Leptospira* of subclades P1 and P2 in soils, Neg_S: soil with no detection of pathogenic *Leptospira* contamination, Si_ro: observation of rodents in paddocks, No_ro: no observation of rodents in paddock, Si_can: observation of canines in paddocks, No_can: no observation of canines in paddocks, Sej_per: seropositivity to Sejroe serogroup in humans, Ict_pers: seropositivity to Icterohaemorrhagiae serogroup in humans, Aut_pers: seropositivity to Autumnalis serogroup in humans, NA_pers: seronegativity to *Leptospira* in humans, MAT_Pos: seropositivity to Leptospira in cattle, MAT_Neg: seronegativity to *Leptospira* in cattle, BATA: seropositivity to Bataviae serogroup in cattle, SANJ: seropositivity to indeterminate serogroup (sanjuanensis) in cattle, POM: seropositivity to Pomona serogroup in cattle, ICT: seropositivity to Icterohaemorrhagiae serogroup in cattle, BAL: seropositivity to Ballum serogroup in cattle, SEJ: seropositivity to Sejroe serogroup in cattle, GRI: seropositivity to Grippotyphosa serogroup in cattle, MIN: seropositivity to Mini serogroup in cattle, TAR: seropositivity to Tarassovi serogroup in cattle, SEMA: seropositivity to Semaranga serogroup in cattle, Mix: mixed seropositivity in cattle, Neg_SEG: seronegativity to Leptospira in cattle, L_Borg: infection by *L. borgpetersenii* in cattle, Neg_PCR: no detection of *Leptospira* infection in cattle, 1_EA: < 1 year of age in cattle, 2_EA: > 1 to 4 years of age in cattle, 3_EA: > 4 to 7 years of age in cattle, 4_EA: > 7 to 10 years of age in cattle, 5_EA: > 10 years of age in cattle, ND_EA: age of cattle without data, HL:growing heifer, CM: bull calf, CH: heifer calf, NV: replacement heifer, VS: dry cow, VP: recently calved cow, Toro: bull, ND_ER: reproductive status of cattle without data, Si_V: vaccination against *Leptospira* in cattle, No_V: absence of vaccination against *Leptospira* in cattle, Si_IB: use of inputs and biosecurity measures in cattle management, No_IB: absence of inputs and biosecurity measures in cattle management, MX_prr: mixed seropositivity in canines, Can_Aut: seropositivity to Autumnalis and Canicola serogroups in canines, Ict_prr: seropositivity to Icterohaemorrhagiae serogroup in canines, Neg_prr: seronegativity to *Leptospira* in canines.

This analysis enabled associations to be identified between the various outcomes evaluated in this study. These included seropositivity, titers, serogroups, and infection in bovines. The analysis also considered various productive and reproductive variables. Furthermore, the results of contamination of water sources and soils were taken into account. The analysis considered the absolute frequency of *Leptospira* seropositivity in humans and canines on each farm. This analysis evaluated the associations between the outcomes and the different landscape metrics related to forest or dense vegetation cover and TWI (areas with TWI > 10). Nearest neighbor analysis was used to calculate a Euclidean distance on the factorial plane, ranging from 0.00 to 12.35. According to these distances, the categories were then determined to be: strongly associated when the distance was between 0.0 and 2.5; moderately similar when the distance was between 2.5 and 5.0; different when the distance was between 5.0 and 8.0; and very different or opposite when the distance was between 8.0 and 12.35. This analysis revealed that the first two dimensions together accounted for 25.3% of the total variability in the dataset, with Dimension 1 contributing 15.7% and Dimension 2 contributing 9.6% (Fig 5).

In this regard, the qualitative categories that contributed most to dimension 1 were the presence of canines and *Leptospira* contamination in water sources. As described in the Methods section, landscape variables categorized as forest or dense vegetation edges, very high length, very high forest fragmentation, very high patch density, and very complex landscape shapes also contributed significantly. Other significant contributors were the categories of high edge length, high fragmentation, high vegetation patch density, and seropositivity in humans against the Sejroe serogroup (S14 Fig). The contribution percentages of these categories to the dimension ranged from 3.6% to 1.04%.

The qualitative categories that contributed most to Dimension 2 were high forest fragmentation (a categorized variable), the absolute frequency of seropositivity in humans against the Icterohaemorrhagiae serogroup and pasture rotation at three-day intervals. Other significant contributors were the very small size of forest or dense vegetation areas, highly floodable areas (including “very highly floodable”) and low or medium forest coverage. Variables with low contributions include the absolute frequency of seropositivity in humans against the Sejroe serogroup and in canines against Icterohaemorrhagiae, the presence of breeding females and bovines vaccinated against *Leptospira*, and positive for *Leptospira* soil samples. Finally, the following demographic and management variables were highlighted: cattle aged 1–4 years, pasture rotation every seven days, and the absolute frequency of seronegatives. The contributions of these categories ranged from 7.07% to 1.10% (S14 Fig).

Regarding the quantitative variables analyzed in the landscape metrics, the variables that contributed most to Dimension 1 were total edge area, patch density, landscape shape index, number of patches, edge density, total dense vegetation area, and proportion of *Leptospira* seropositive in humans per farm. Their respective contributions ranged from 6.7% to 1.05% (S15 Fig). Similarly, the variables that contributed most to Dimension 2 were the largest patch index, the topographic wetness index, the proportion of the landscape occupied by forest or dense vegetation, total area of forest or dense vegetation, the proportion of *Leptospira* seropositive in humans per farm and edge density. Their contributions ranged from 9.4% to 1.05% (S16 Fig).

**Fig 6:**
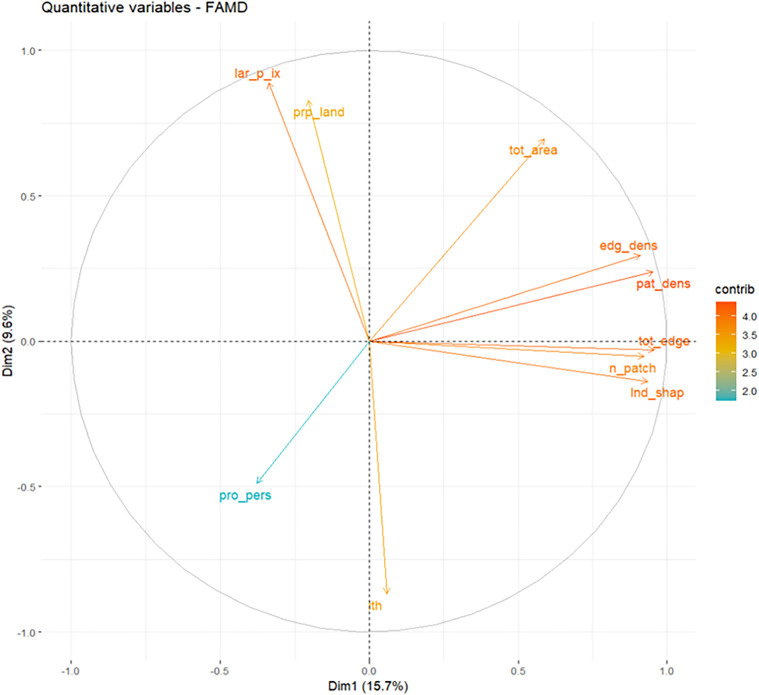
Contribution of quantitative variables in the MDFA. tot_area: total dense vegetation area, prp_land: proportion of dense vegetation area within the landscape, edg_dens: edge density, pat_dens: patch density, tot_edge: total edge, n_patch: number of patches, lar_p_ix: largest patch index, lnd_shap: landscape shape index, ith: topographic wetness index, pro_pers: proportion of seropositivity in humans.

Based on the analysis of nearby neighbors, several highly associated categories were identified that contributed significantly to the main dimensions of the analysis. Seropositivity against the Bataviae serogroup with titres of 1:400 in bovines was associated with the presence of canines and rodents, the detection of *Leptospira* in water samples, and high fragmentation, density, and edge length, as well as very complex dense vegetation morphology (Fig 5, ellipse 1). Furthermore, seropositivity against the Bataviae serogroup with titres of 1:400 in bovines was associated with quantitative variables such as the number of patches, shape index, total edge length and patch and edge densities (Fig 6).

Conversely, seropositivity with titres of 1:1600 against the Ballum serogroup, 1:400 against the Grippotyphosa serogroup, 1:3200 against the Tarassovi serogroup, and 1:200 against the Icterohaemorrhagiae serogroup in bovines was associated with the absolute frequency of seropositivity against the Sejroe serogroup in humans, as well as with the presence of male offspring (Fig 5, ellipse 2). Similarly, seropositivity with titres of 1:1,600 against the Mini serogroup, 1:800 against the Bataviae serogroup, and 1:400 and 1:800 against the Sejroe serogroup in cattle was associated with *L. borgpetersenii* infection in cattle, the absolute frequency of seropositivity against the Icterohaemorrhagiae serogroup in humans, and quantitative variables such as landscape proportion and largest patch index (Fig 5, ellipse 3, and Fig 6).

Seropositivity with titres of 1:100 against the Pomona serogroup and 1:200 and 1:800 against the Icterohaemorrhagiae serogroup in cattle was associated with a small area of forest or dense vegetation, soil contaminated by P1 and P2 subclades of pathogenic *Leptospira*, cattle vaccination and the proportion of seropositive humans (quantitative variable) (Fig 5, ellipse 4, and Fig 6).

Conversely, seropositivity against the Canicola and Autumnalis serogroups in canines was associated with the absolute frequency of seropositivity against the Autumnalis serogroup in humans. It was also associated with the presence of pathogenic leptospires in the soil and a very high topographic wetness index (flooding category). Additionally, it was associated with highly fragmented forests (categorized variable) (Fig 5, ellipse 5).

Lastly, seropositivity with titres of 1:400 against *Leptospira sanjuanensis*, as well as mixed seropositivity with titres of 1:1,600 in cattle, was associated with seropositivity in canines to serogroup Icterohaemorrhagiae, heifers in the reproductive category, and the total area of forest or dense vegetation (Fig 5, ellipse 6).

## Discussion

A One Health approach was conducted to study the relationship between infections, leptospiras contamination in the environment, and landscape composition in humans, cattle, and canines. The proportion of *Leptospira* seropositivity in cattle was calculated to be 76.9%. In our study, samples with titres of 1:100 or above were classified as seropositive. This criterion was also employed in a 2014 study conducted in San Pedro de los Milagros, Antioquia. The study reported a proportion of *Leptospira* seropositivity of 12.4%, primarily against the Sejroe, Pomona, Grippotyphosa, and Tarassovi serogroups [23]. The same outcome measure has been used in other studies. For instance, Orjuela et al. [24] identified *Leptospira* antibodies in 41.8% of cattle on the north coast of Colombia. Furthermore, seroprevalence of 80.5% [25] and 49% [26] were reported in Venezuela and the United States, respectively.

To date, no studies have reported on *Leptospira* infection or seropositivity in cattle in the Urabá region. Therefore, the high seropositivity observed in this study may be related to the region’s specific conditions including management practices, ecological factors, environmental conditions and limited access to preventive measures typical of extensive beef production.

Of the serogroups identified in cattle, Tarassovi was the most frequent in our study, accounting for 31.3% of cases. This finding is consistent with results obtained in Brazil (22.2%) [27]. These findings suggest an established exposure to this serogroup in the local ecosystem, potentially linked to the presence of hosts such as pigs or rodents [28]. In this study, serogroup Mini was the second most frequently identified in cattle, accounting for 30.2% of cases. It has previously been reported as the most prevalent serogroup in cattle in India (36.2%) [29]. In Colombia, this serogroup has been identified in pigs, canine and humans [30]. The Ballum serogroup was found in 14.47% of seropositive cattle in this study. It is primarily recognized as having rodents as its main host, and as one of the serogroups most frequently excreted in the urine of these animals [31]. A particularly relevant finding was the low seropositivity to *L. sanjuanensis*, a pathogenic subclade 1 species recently identified in soil samples in Puerto Rico. However, its reservoir and the associated risks of disease and transmission remain unknown [32]. As this is a recently described species, it is important to note that its inclusion as an antigen in the microscopic agglutination test (MAT) has scarcely been evaluated. Although this species has not been previously reported in Colombia, its serological detection could indicate cross-reactivity with related serogroups or the presence of the species in the region going unnoticed.

In light of the aforementioned findings, the results of this study differ from those typically reported in the literature. These studies have described the Sejroe serogroup, which includes the Hardjo prajitno and Hardjo bovis serovars belonging to the *L. interrogans* and *L. borgpetersenii* species, as the most prevalent in cattle [33].

Regarding serological titres, 7.11% of seropositive animals had titres of 1:1600 or greater, which suggests possible recent exposure to the bacterium in the absence of vaccination [34] However, since only the animals on Farm 1 were vaccinated and three of these animals had titres of at least 1:1600 seven months after vaccination, which is consistent with the persistence of high titres induced by the vaccine (≥1:400) for four to six months [35], it is reasonable to conclude that these cattle were recently exposed to *Leptospira*. A sensitivity analysis was performed to evaluate the possible effect of vaccination on this farm. The results of this analysis were consistent with those of the study. By contrast, the infected cattle in this study had titres lower than 1:800. However, Silva et al. [36] reported a high probability of *Leptospira* elimination when antibodies against *Leptospira* with titers of ≥1:1600 were detected.

Identification of *L. borgpetersenii* is consistent with the primary serogroups identified in cattle: Tarassovi, Mini, and Ballum. This finding is similar to a study by Hamond et al. [37], who isolated *L. borgpetersenii* serogroup Tarassovi from cow urine in the United States. This suggests that *L. borgpetersenii* may act as a source of infection for other animals or as an environmental contaminant.

A proportion of *Leptospira* seropositivity of 33.3% was found in canines against the Canicola, Icterohaemorrhagiae, and Autumnalis serogroups. These serogroups are all potentially related to *L. interrogans*. In a study conducted in Antioquia, reported that Urabá had the highest frequency of *Leptospira* seropositivity cases, specifically against the Canicola and Icterohaemorrhagiae serogroups. This study also found that untreated water sources were associated with a 2.2-fold higher frequency of seropositive canines, as well as higher seropositivity in homes with inadequate waste disposal [38].

Previous studies have reported seropositivity against serogroups such as Canicola, Icterohaemorrhagiae, Bataviae, and Tarassovi in various species, including dogs, humans, rodents, and pigs. These studies have also detected pathogenic *Leptospira* in water sources [30,39]. These results support the hypothesis that domestic and synanthropic animals play a role in both eliminating the bacteria and contaminating the environment. Similarly, seropositivity against the Autumnalis serogroup has been reported in dogs, pigs, and humans [30]. Together, these findings corroborate the results of this study, which revealed an association between the presence of canines and rodents, and the detection of pathogenic leptospires in water sources. Additionally, a relationship was found between canines and humans who were seropositive for the Autumnalis serogroup. The presence of the Autumnalis serogroup in both humans and dogs within the same farm suggests shared exposure or transmission within the home or peridomestic environment. Besides, poorly cared-for canines can act as amplifying hosts, making them a relevant link in the chain of transmission risk for people living with them [40–42]. For instance, a study in Bhutan examined risk factors for *Leptospira* exposure in canines, humans, and livestock. The study suggested that dogs could be a source of human infection or that both species could be exposed to the same environmental risk factor [43].

The present study found that 4.1% of people were seropositive for *Leptospira*. However, studies conducted in the same region have reported seroprevalence of 27.81% [44]. Other studies have reported a proportion of *Leptospira* seropositivity of 58.0% [45] and 12.5% [46].

It is important to emphasize that people who work indoors are exposed to *Leptospira.* Romero-Vivas et al. [39] found that the majority of patients confirmed to have leptospirosis in Barranquilla were housewives, unemployed individuals or students. However, several authors have pointed out that the risk of human infection increases in outdoor occupations [44]. In this regard, Taddei et al. [47] suggested that, as potential hosts that spread leptospires, cattle pose a relatively lower risk of direct infection to humans than other reservoirs, such as rodents or canines. This highlights the importance of assessing local transmission dynamics and promoting prevention measures targeting not only agricultural workers, but also their household members.

The *Leptospira* serogroups identified in this study were Icterohaemorrhagiae, Autumnalis, and Sejroe, consistent with previous reports from different regions of Urabá. The potential circulation of the following serogroups is also suggested: Bataviae, Tarassovi, Djasiman, Icterohaemorrhagiae, Pyrogenes, Panama, Cynopteri, Autumnalis, Grippotyphosa and Mini [44]. However, the importance of the public health surveillance system in identifying different serogroups in the region must be highlighted. The ability to detect these serogroups may vary according to the strains used for diagnosis or the presence of different eco-epidemiological conditions affecting the transmission of these agents. Serogroup Icterohaemorrhagiae is strongly associated with synanthropic rodents, whereas Sejroe is more prevalent in production animals, such as cattle. This suggests different exposure routes in different contexts [48]. During 2024, the National Institute of Health (INS) [49] reported the presence of other serogroups in confirmed cases of leptospirosis: Andamana, Australis and Pomona. These differences may be due to the diagnostic panels used, reflecting greater serological diversity in the Antioquia department. It is also important to evaluate the relationship between *Leptospira* transmission in rural environments and outdoor activities. Three of the seropositive individuals in this study lived in rural areas and traveled through forests, pastures, and ravines every day on their way to work. Furthermore, two of these individuals reported frequently consuming water from natural sources. Several studies have reported an association between exposure to *Leptospira* and rural environments [5], as well as contact with natural water sources and recreational water activities. Other associated factors include exposure to flooding, mud and stagnant water. These factors increase the likelihood of coming into contact with these infectious agents [50].

This study identified the presence of leptospires that are more closely related to *L. tipperaryensis*, a species belonging to pathogenic subclade 1. This species was initially isolated in grey shrews (*Crocidura russula*), particularly in areas where it displaced the pygmy shrew (*Crocidura suaveolens*) in Ireland [51]. The bacterium has also been reported in rats (*Rattus norvegicus*) in France [52]. In the same study, water sources used for recreational activities in the summer were sampled, and *L. tipperaryensis* was not detected in these waters.

This study also found an association between *L. tipperaryensis* contamination of water and the presence of rodents and highly fragmented forest areas. In such areas, biodiversity is reduced and native species may be displaced by exotic species or rodents [53]. The detection of this agent also suggests that it may persist in environmental reservoirs and participate in zoonotic cycles. Although its direct involvement in human cases remains to be demonstrated, its classification within pathogenic subclade 1 highlights the need for further research to improve our understanding of its role in *Leptospira* epidemiology and disease.

Regarding to the detection of potentially pathogenic *Leptospira* in soil, agents that are phylogenetically related to *L. weili*i and *L. cinconiae* were identified. *L. weilii* has been found in several species, including humans, cattle, pigs, canines, and rodents. Its ability to persist in water and soil has been documented [54]. *Leptospira cinconiae* was recently isolated from water samples in Iowa, United States [55]. The identification of this species, particularly *L. weilii*, emphasises the significance of environmental reservoirs in the transmission of *Leptospira* and the potential risk to humans and other animals. Also, this study revealed an association between contaminated soil and seropositivity in cattle to the Pomona and Icterohaemorrhagiae serogroups, which are generally linked to pigs and rodents, respectively [56].

This study found that areas on farms with a high probability of flooding were associated with highly fragmented forest areas, as well as seropositivity to the Canicola serogroup in canines and the Autumnalis serogroup in both dogs and humans. Other studies have reported this relationship, indocating that flooding events are more frequent in deforested areas or areas with degraded forests [57]. Similarly, exposure to *Leptospira* has been shown to increase in flood-prone areas, affecting humans and domestic animals [58]. Some hypotheses suggest that pathogenic leptospires can persist in soil and be washed into surface waters during heavy rainfall. There, they remain in suspension, thus increasing the risk of infection [59].

Although this study was conducted during the dry season, the detection of pathogenic leptospires in lakes indicates that these bacteria can survive in the environment, even without recent rainfall. This implies that various animals, both domestic and wild, could become contaminated or infected by coming into contact with these water sources.

Regarding the landscape, several authors report that the measures used in this study variables are used to assess fragmentation and configuration, particularly the presence of dense vegetation or forests, to evaluate conservation status and habitat connectivity [14]. This plays a fundamental role in exposure to different animal species and environmental contamination. In a study conducted in Rio Grande do Sul, Brazil, determined that decreased forest cover and increased edge density were landscape factors associated with primate exposure to *Leptospira*. This indicates that habitat fragmentation can increase contact with pathogen [60]. However, other studies have identified different risk factors. For instance, a study conducted in Necoclí, Antioquia, identified areas with forest cover of more than 10% as risk factors, reporting that people working from home were more exposed to *Leptospira* when dense vegetation cover was high. These authors hypothesized that this association could be mediated by vegetation-induced microclimatic patterns [61]. Another study in the Amazon region suggested that deforestation in the area may have contributed to the presence of *Leptospira*-seropositive canines, as high forest fragmentation decreases biodiversity and reduces the dilution effect [62].

This phenomenon, whereby high species diversity reduces the risk of disease transmission, has been documented in leptospirosis and other infectious diseases such as West Nile virus and Lyme disease [63]. In the case of *Leptospira*, loss of biodiversity allows generalist or invasive species, such as synanthropic rodents, to proliferate, while reducing the presence of natural predators. This increases the circulation of the pathogen in the environment [53]. A recent study in Madagascar supports this hypothesis, showing that habitats with significant human-caused changes have a higher relative abundance of exotic species. A significantly higher prevalence of *Leptospira* was recorded in these exotic species compared to native species. Additionally, a lower probability of infection was found in unfragmented forests [64].

Additionally, it is important to recognize how land use and landscape characteristics could modify the ecological composition of small mammal communities and the dynamics of disease transmission. Similarly, it is important to recognize how the proliferation of rodents could facilitate direct and indirect contact between these reservoirs and other domestic animal species, such as canines and cattle. This includes contact with humans, particularly in rural areas where there are structural deficiencies and high rates of unmet basic needs.

The results of this study raise several relevant research questions regarding cattle, human and canine populations, as well as the environment. For example, which species of domestic, wild, or synanthropic animals participate in the infection cycle of cattle, particularly with regard to *L. borgpetersenii*? Are there spatial or temporal patterns in bovine infections that identify areas or periods of higher risk? Which mammalian hosts are associated with *Leptospira sanjuanensis*? Are there other species of domestic, wild, or synanthropic animals involved in the canine infection cycle? Could a public health surveillance system for leptospirosis be implemented based on the One Health approach?

On the other hand, the following questions were generated for the study of the environmental component related to the transmission of infectious agents: Which animals (domestic, wild, and synanthropic) primarily contaminate environmental sources with *Leptospira*? Does low biodiversity resulting from forest fragmentation constitute a risk factor for *Leptospira* infection in this region? How can forest fragmentation be addressed to reduce the potential risk of *Leptospira* transmission to people and domestic animals in the Urabá region?

This study has limitations that must be considered when interpreting the results. Firstly, probabilistic sampling of cattle was initially proposed but this could not be implemented due to logistical constraints. This restricts the ability to conduct inferential analyses on the local population and establish causal relationships between the evaluated variables. Additionally, the sample sizes for humans and canines were relatively small (n = 73 and n = 15, respectively), which may have affected the representativeness of the findings for these groups. Furthermore, some cattle and canines had a history of vaccination, making it difficult to distinguish between antibodies generated by vaccination and those produced by natural infection, particularly in cases with intermediate serological titres.

Administrative issues also arose during data collection that affected data integrity. In particular, information relating to eight cattle was lost because the identification number reported at the time of sampling did not correspond to any official record in the software. Nevertheless, these animals were included in the analyses because serum and urine samples were available for them. Similarly, while all participants completed the personal survey, specific housing data could not be collected for seven of them, limiting the analysis to the family level.

When it comes to identifying the etiological agent, interpreting serological results is inherently limitations. Although the MAT is considered the gold standard for diagnosis, cross-reactions between different serogroups can occur, making it difficult to identify the infecting serogroup. Additionally, the subjective nature of positivity determination in this test, which depends on visual interpretation of the degree of agglutination, introduces variability between observers. This limitation is well known in *Leptospira* studies and must be considered when interpreting results.

Finally, although areas occupied by armed groups were excluded, it was not possible to fly a drone over Farm 7 for security reasons. Therefore, images were obtained using satellite sensors instead. This resulted in a difference in spatial resolution (pixel size) of the images, which may have impacted the accuracy of the landscape indicator estimates compared to those of the other farms and potentially biasing the results. Despite these limitations, the study still provides valuable insights into *Leptospira* exposure in the Urabá region and highlights the importance of landscape characteristics in the transmission cycle among animals and humans.

In conclusion, the study identified a high proportion of *Leptospira* seropositivity in cattle, with multiple serogroups present. Contaminated environmental samples (water and soil) were also found, indicating the presence of the pathogen in the environment.

No correlation was found between *Leptospira* species in animals, humans and the environment, suggesting simultaneous but independent transmission cycles. The study also revealed a potential epidemiological link between dogs and humans, as well as a distinct transmission cycle in cattle possibly linked to alternative reservoirs.

An association was found between seropositivity and landscape variables, particularly forest fragmentation and composition. These conditions could favour the persistence of the pathogen in the environment and its circulation among hosts.

From a One Health perspective, these findings can help guide future studies strategies in the region and in vulnerable rural areas. It is recommended that the sample size is increased, probabilistic sampling is included, and the study is extended to include other reservoir species. Additionally, it is suggested that diagnostic techniques be complemented with culture isolation and that the role of new *Leptospira* species recently identified in the region be explored.

## Supporting information

S1 Table Confusion matrix of random forest model. (DOCX)

S2 Table Description of the landscape metrics for Farm 1. (DOCX)

S3 Table Description of the landscape metrics for Farm 3. (DOCX)

S4 Table Description of the landscape metrics for Farm 4. (DOCX)

S5 Table Description of the landscape metrics for Farm 5. (DOCX)

S6 Table Description of the landscape metrics for Farm 6. (DOCX)

S7 Table Description of the landscape metrics for Farm 7. (DOCX)

S8 Table Dataset for information analysis in MDFA. (XLSX)

S1 Fig Orthomosaic and land classification of the Farm 1. (A) Orthomosaic of the Farm 1. (B) Land classification of the Farm 1, showing: pastures (light green), dense vegetation (dark green), water bodies (blue) and built-up areas (red). The base map was developed through the manual delineation of farm boundaries by the research team. The map was created in QGIS software.
(DOCX)

S2 Fig Orthomosaic and land classification of the Farm 3. (A) Orthomosaic of the Farm 3. (B) Land classification of the Farm 3, showing: pastures (light green), dense vegetation (dark green), water bodies (blue) and built-up areas (red). The base map was developed through the manual delineation of farm boundaries by the research team. The map was created in QGIS software.
(DOCX)

S3 Fig Orthomosaic and land classification of the Farm 4. (A) Orthomosaic of the Farm 4. (B) Land classification of the Farm 4, showing: pastures (light green), dense vegetation (dark green), water bodies (blue) and built-up areas (red). The base map was developed through the manual delineation of farm boundaries by the research team. The map was created in QGIS software.
(DOCX)

S4 Fig Orthomosaic and land classification of the Farm 5. (A) Orthomosaic of the Farm 5. (B) Land classification of the Farm 5, showing: pastures (light green), dense vegetation (dark green), water bodies (blue) and built-up areas (red). The base map was developed through the manual delineation of farm boundaries by the research team. The map was created in QGIS software.
(DOCX)

S5 Fig Orthomosaic and land classification of the Farm 6. (A) Orthomosaic of the Farm 6. (B) Land classification of the Farm 6, showing: pastures (light green), dense vegetation (dark green), water bodies (blue) and built-up areas (red). The base map was developed through the manual delineation of farm boundaries by the research team. The map was created in QGIS software.
(DOCX)

S6 Fig Orthomosaic and land classification of the Farm 7. (A) Orthomosaic of the Farm 7. (B) Land classification of the Farm 7, showing: pastures (light green), dense vegetation (dark green), water bodies (blue) and built-up areas (red). The base map was developed through the manual delineation of farm boundaries by the research team. The map was created in QGIS software.
(DOCX)

S7 Fig Digital elevation model and flooded area of the Farm 1. (A) Digital elevation model of the Farm 1. (B) Flooded area of the Farm 1. (C) Proportion of floodable area of the Farm 1. The base map was developed through the manual delineation of farm boundaries by the research team. The map was created in QGIS software.
(DOCX)

S8 Fig Digital elevation model and flooded area of the Farm 3. (A) Digital elevation model of the Farm 3. (B) Flooded area of the Farm 3. (C) Proportion of floodable area of the Farm 3. The base map was developed through the manual delineation of farm boundaries by the research team. The map was created in QGIS software.
(DOCX)

S9 Fig Digital elevation model and flooded area of the Farm 4. (A) Digital elevation model of the Farm 4. (B) Flooded area of the Farm 4. (C) Proportion of floodable area of the Farm 4. The base map was developed through the manual delineation of farm boundaries by the research team. The map was created in QGIS software.
(DOCX)

S10 Fig Digital elevation model and flooded area of the Farm 5. (A) Digital elevation model of the Farm 5. (B) Flooded area of the Farm 5. (C) Proportion of floodable area of the Farm 5. The base map was developed through the manual delineation of farm boundaries by the research team. The map was created in QGIS software.
(DOCX)

S11 Fig Digital elevation model and flooded area of the Farm 6. (A) Digital elevation model of the Farm 6. (B) Flooded area of the Farm 6. (C) Proportion of floodable area of the Farm 6. The base map was developed through the manual delineation of farm boundaries by the research team. The map was created in QGIS software.
(DOCX)

S12 Fig Digital elevation model and flooded area of the Farm 7. (A) Digital elevation model of the Farm 7. (B) Flooded area of the Farm 7. (C) Proportion of floodable area of the Farm 7. The base map was developed through the manual delineation of farm boundaries by the research team. The map was created in QGIS software.
(DOCX)

S13 Fig Contribution of qualitative variables to dimension 1 in the MDFA. Si_can: presence of canines in paddocks, P1_A: contamination by Leptospira of subclade P1 in water sources, MA_longB: very high edge length, MA_frag: very high fragmentation, MA_den_P: very high patch density, F_Mcomp: very complex landscape shape, Si_ro: presence of rodents in paddocks, F_comp: complex landscape shape, A_longB: high edge length, A_frag: high fragmentation, A_den_P: high patch density, Sej_per: seropositivity to Sejroe serogroup in humans, No_can: absence of canines in paddocks, A_den_B: high edge density, NA_pers: seronegativity to Leptospira in humans, MA_den_B: very high edge density, Neg_A: water with no detection of pathogenic Leptospira contamination, B_rep: low representation of dense vegetation area in the landscape, < 7: paddock rotation every 7 days, MX_prr: mixed seropositivity in canines, Ict_pers: seropositivity to Icterohaemorrhagiae serogroup in humans, < 3: paddock rotation every 3 days, M_peq: very small dense vegetation area, M_rep: medium representation of dense vegetation area in the landscape, 1_EA: < 1 year of age in cattle.
(DOCX)

S14 Fig Contribution of qualitative variables to dimension 2 in the MDFA. Frag: largest fragmented patch, Ict_pers: seropositivity to Icterohaemorrhagiae serogroup in humans, < 3: paddock rotation every 3 days, M_peq: very small dense vegetation area, A_inun: highly floodable landscape, M_frag: largest highly fragmented patch, Peq: small dense vegetation area, MA_inun: very highly floodable landscape, B_rep: low representation of dense vegetation area in the landscape, M_rep: medium representation of dense vegetation area in the landscape, Sej_per: seropositivity to Sejroe serogroup in humans, Ict_prr: seropositivity to Icterohaemorrhagiae serogroup in canines, HL:growing heifer, Si_V: vaccination against Leptospira in cattle, P1_P2_S: contamination by Leptospira of subclades P1 and P2 in soils, 2_EA: > 1–4 years of age in cattle, < 7: paddock rotation every 7 days, Neg_prr: seronegativity to Leptospira in canines, MX_prr: mixed seropositivity in canines, 1_EA: < 1 year of age in cattle, Can_Aut: seropositivity to Autumnalis and Canicola serogroups in canines, Aut_pers: seropositivity to Autumnalis serogroup in humans, CM: bull calf, L_Borg: infection by L. borgpetersenii in cattle, CH: heifer calf.
(DOCX)

S15 Fig Contribution of quantitative variables to dimension 1 in the MDFA. tot_edge: total edge, pat_dens: patch density, lnd_shap: landscape shape index, n_patch: number of patches, edg_dens: edge density, tot_area: total dense vegetation area, pro_pers: proportion of seropositivity in humans, lar_p_ix: largest patch index, prp_land: proportion of dense vegetation area within the landscape, ith: topographic wetness index.
(DOCX)

S16 Fig Contribution of quantitative variables to dimension 2 in the MDFA. lar_p_ix: largest patch index, ith: topographic wetness index, prp_land: proportion of dense vegetation area within the landscape, tot_area: total dense vegetation area, pro_pers: proportion of seropositivity in humans, edg_dens: edge density, pat_dens: patch density, lnd_shap: landscape shape index,n_patch: number of patches, tot_edge: total edge.
(DOCX)
