# Supplementary material for: Epidemiological study of leptospiral interaction in bovine farms in rural areas of Colombia: A One Health approach
Source: PLoS Negl Trop Dis. 2026 May 6;20(5):e0014231. doi: 10.1371/journal.pntd.0014231 (PMC13170971; doi:10.1371/journal.pntd.0014231)
Supplement: S1 Table — (DOCX) [file pntd.0014231.s001.docx]

**S1 Table. Confusion matrix of Random Forest model.**

**Farm 1**

| **Image 1** | | | | | |
| --- | --- | --- | --- | --- | --- |
| **Confusion matrix** | | | | | |
| [[295643 | 2646 | 310 | 0 | 0 | 45] |
| [20444 | 19225 | 187 | 5 | 4 | 154] |
| [175 | 122 | 15077 | 4 | 0 | 2] |
| [6 | 0 | 7 | 3389 | 0 | 0] |
| [39 | 19 | 0 | 0 | 0 | 0] |
| [825 | 951 | 13 | 1 | 0 | 97]] |
|  | | | | | |
| **Classification report** | | | | | |
|  | **Precision** | **Recall** | **F1-score** | **Support** | |
| 1 | 0.93 | 0.99 | 0.96 | 298644 | |
| 2 | 0.84 | 0.48 | 0.61 | 40019 | |
| 3 | 0.97 | 0.98 | 0.97 | 15380 | |
| 4 | 1.00 | 1.00 | 1.00 | 3402 | |
| 5 | 0.00 | 0.00 | 0.00 | 58 | |
| 6 | 0.33 | 0.05 | 0.09 | 1887 | |
|  | | | | | |
| **Accuracy** |  |  | 0.93 | 359390 | |
| **Macro avg** | 0.68 | 0.58 | 0.60 | 359390 | |
| **Weighted avg** | 0.92 | 0.93 | 0.92 | 359390 | |

| **Image 2** | | | | |
| --- | --- | --- | --- | --- |
| **Confusion matrix** | | | | |
| [[368311 | 1872 | 0 | 0 | 5] |
| [26848 | 10326 | 26 | 0 | 2] |
| [5814 | 11 | 9412 | 0 | 0] |
| [2160 | 0 | 0 | 69 | 0] |
| [1112 | 20 | 0 | 0 | 0]] |
|  | | | | |
| **Classification report** | | | | |
|  | **Precision** | **Recall** | **F1-score** | **Support** |
| 1 | 0.91 | 0.99 | 0.95 | 370188 |
| 2 | 0.84 | 0.28 | 0.42 | 37202 |
| 3 | 1.00 | 0.62 | 0.76 | 15237 |
| 4 | 1.00 | 0.03 | 0.06 | 2229 |
| 6 | 0.00 | 0.00 | 0.00 | 1132 |
|  | | | | |
| **Accuracy** |  |  | 0.91 | 425988 |
| **Macro avg** | 0.75 | 0.38 | 0.44 | 425988 |
| **Weighted avg** | 0.91 | 0.91 | 0.89 | 425988 |

| **Image 3** | | | | |
| --- | --- | --- | --- | --- |
| **Confusion matrix** | | | | |
| [[294420 | 1264 | 1 | 0 | 11] |
| [35713 | 6560 | 11 | 0 | 12] |
| [9548 | 9 | 383 | 0 | 0] |
| [17 | 0 | 0 | 59 | 0] |
| [533 | 72 | 0 | 0 | 14]] |
|  | | | | |
| **Classification report** | | | | |
|  | **Precision** | **Recall** | **F1-score** | **Support** |
| 1 | 0.87 | 1.00 | 0.93 | 295696 |
| 2 | 0.83 | 0.16 | 0.26 | 42296 |
| 3 | 0.97 | 0.04 | 0.07 | 9940 |
| 4 | 1.00 | 0.78 | 0.87 | 76 |
| 6 | 0.38 | 0.02 | 0.04 | 619 |
|  | | | | |
| **Accuracy** |  |  | 0.86 | 348627 |
| **Macro avg** | 0.81 | 0.40 | 0.44 | 348627 |
| **Weighted avg** | 0.86 | 0.86 | 0.82 | 348627 |

**Farm 2**

| **Image 1** | | | | |
| --- | --- | --- | --- | --- |
| **Confusion matrix** | | | | |
| [[1231397 | 25576 | 0 | 0 | 2] |
| [192119 | 214234 | 37 | 0 | 7] |
| [41205 | 42 | 12754 | 0 | 0] |
| [0 | 0 | 0 | 749 | 0] |
| [2 | 5 | 0 | 0 | 66]] |
|  | | | | |
| **Classification report** | | | | |
|  | **Precision** | **Recall** | **F1-score** | **Support** |
| 1 | 0.84 | 0.98 | 0.90 | 1256975 |
| 2 | 0.89 | 0.53 | 0.66 | 406397 |
| 3 | 1.00 | 0.24 | 0.38 | 54001 |
| 4 | 1.00 | 1.00 | 1.00 | 749 |
| 5 | 0.88 | 0.90 | 0.89 | 73 |
|  | | | | |
| **Accuracy** |  |  | 0.85 | 1718195 |
| **Macro avg** | 0.92 | 0.73 | 0.77 | 1718195 |
| **Weighted avg** | 0.86 | 0.85 | 0.83 | 1718195 |

| **Image 2** | | | | |
| --- | --- | --- | --- | --- |
| **Confusion matrix** | | | | |
| [[2226657 | 62551 | 322 | 286 | 1] |
| [538721 | 382066 | 930 | 373 | 18] |
| [11978 | 378 | 159734 | 701 | 0] |
| [14439 | 197 | 2277 | 8747 | 0] |
| [99 | 604 | 0 | 0 | 7]] |
|  | | | | |
| **Classification report** | | | | |
|  | **Precision** | **Recall** | **F1-score** | **Support** |
| 1 | 0.80 | 0.97 | 0.88 | 2289817 |
| 2 | 0.86 | 0.41 | 0.56 | 922108 |
| 3 | 0.98 | 0.92 | 0.95 | 172791 |
| 4 | 0.87 | 0.34 | 0.49 | 25660 |
| 5 | 0.27 | 0.01 | 0.02 | 710 |
|  | | | | |
| **Accuracy** |  |  | 0.81 | 3411086 |
| **Macro avg** | 0.75 | 0.53 | 0.58 | 3411086 |
| **Weighted avg** | 0.82 | 0.81 | 0.79 | 3411086 |

| **Image 3** | | | | |
| --- | --- | --- | --- | --- |
| **Confusion matrix** | | | | |
| [[2641259 | 70354 | 555 | 327 | 0] |
| [411011 | 316556 | 216 | 199 | 0] |
| [115362 | 218 | 13963 | 99 | 0] |
| [6097 | 115 | 235 | 15237 | 0] |
| [50 | 0 | 0 | 0 | 0]] |
|  | | | | |
| **Classification report** | | | | |
|  | **Precision** | **Recall** | **F1-score** | **Support** |
| 1 | 0.83 | 0.97 | 0.90 | 2712495 |
| 2 | 0.82 | 0.43 | 0.57 | 727982 |
| 3 | 0.93 | 0.11 | 0.19 | 129642 |
| 4 | 0.96 | 0.70 | 0.81 | 21684 |
| 5 | 0.00 | 0.00 | 0.00 | 50 |
|  | | | | |
| **Accuracy** |  |  | 0.83 | 3591853 |
| **Macro avg** | 0.71 | 0.44 | 0.49 | 3591853 |
| **Weighted avg** | 0.83 | 0.83 | 0.80 | 3591853 |

| **Image 4** | | | | |
| --- | --- | --- | --- | --- |
| **Confusion matrix** | | | | |
| [[2675201 | 39793 | 4898 | 36 | 11] |
| [250993 | 128594 | 767 | 37 | 36] |
| [18143 | 355 | 25017 | 0 | 0] |
| [129 | 42 | 5 | 2150 | 0] |
| [611 | 543 | 4 | 0 | 0]] |
|  | | | | |
| **Classification report** | | | | |
|  | **Precision** | **Recall** | **F1-score** | **Support** |
| 1 | 0.91 | 0.98 | 0.94 | 2719939 |
| 2 | 0.76 | 0.34 | 0.47 | 380427 |
| 3 | 0.82 | 0.57 | 0.67 | 43515 |
| 4 | 0.97 | 0.92 | 0.95 | 2326 |
| 5 | 0.00 | 0.00 | 0.00 | 1158 |
|  | | | | |
| **Accuracy** |  |  | 0.90 | 3147365 |
| **Macro avg** | 0.69 | 0.56 | 0.61 | 3147365 |
| **Weighted avg** | 0.89 | 0.90 | 0.88 | 3147365 |

**Farm 3**

| **Image 1** | | | | |
| --- | --- | --- | --- | --- |
| **Confusion matrix** | | | | |
| [[769287 | 37711 | 977 | 2 | 7] |
| [51791 | 74444 | 1377 | 1 | 11] |
| [8664 | 7355 | 10346 | 0 | 0] |
| [145 | 1426 | 2 | 0 | 0] |
| [239 | 1088 | 8 | 0 | 0]] |
|  | | | | |
| **Classification report** | | | | |
|  | **Precision** | **Recall** | **F1-score** | **Support** |
| 1 | 0.93 | 0.95 | 0.94 | 807984 |
| 2 | 0.61 | 0.58 | 0.60 | 127624 |
| 3 | 0.81 | 0.39 | 0.53 | 26365 |
| 4 | 0.00 | 0.00 | 0.00 | 1573 |
| 5 | 0.00 | 0.00 | 0.00 | 1335 |
|  | | | | |
| **Accuracy** |  |  | 0.89 | 964881 |
| **Macro avg** | 0.47 | 0.39 | 0.41 | 964881 |
| **Weighted avg** | 0.88 | 0.89 | 0.88 | 964881 |

| **Image 2** | | | | |
| --- | --- | --- | --- | --- |
| **Confusion matrix** | | | | |
| [[1757604 | 25209 | 2075 | 52 | 64] |
| [148308 | 58703 | 2669 | 107 | 112] |
| [13699 | 9692 | 14994 | 24 | 48] |
| [1585 | 383 | 94 | 925 | 10] |
| [3159 | 1342 | 246 | 25 | 32]] |
|  | | | | |
| **Classification report** | | | | |
|  | **Precision** | **Recall** | **F1-score** | **Support** |
| 1 | 0.91 | 0.98 | 0.95 | 1785004 |
| 2 | 0.63 | 0.28 | 0.39 | 209899 |
| 3 | 0.75 | 0.42 | 0.54 | 35727 |
| 4 | 0.82 | 0.31 | 0.45 | 2997 |
| 5 | 0.12 | 0.01 | 0.01 | 4804 |
|  | | | | |
| **Accuracy** |  |  | 0.90 | 2038431 |
| **Macro avg** | 0.65 | 0.40 | 0.47 | 2038431 |
| **Weighted avg** | 0.88 | 0.90 | 0.88 | 2038431 |

| **Image 3** | | | | |
| --- | --- | --- | --- | --- |
| **Confusion matrix** | | | | |
| [[2163324 | 4898 | 3107 | 39] | |
| [31381 | 7609 | 300 | 9] | |
| [28188 | 785 | 29489 | 7] | |
| [1317 | 4 | 29 | 754]] | |
|  | | | | |
| **Classification report** | | | | |
|  | **Precision** | **Recall** | **F1-score** | **Support** |
| 1 | 0.97 | 1.00 | 0.98 | 2171368 |
| 2 | 0.57 | 0.19 | 0.29 | 39299 |
| 3 | 0.90 | 0.50 | 0.65 | 58469 |
| 4 | 0.93 | 0.36 | 0.52 | 2104 |
|  | | | | |
| **Accuracy** |  |  | 0.97 | 2271240 |
| **Macro avg** | 0.84 | 0.51 | 0.61 | 2271240 |
| **Weighted avg** | 0.96 | 0.97 | 0.96 | 2271240 |

| **Image 4** | | | | |
| --- | --- | --- | --- | --- |
| **Confusion matrix** | | | | |
| [[1579290 | 2999 | 1357 | 82] | |
| [30257 | 3985 | 174 | 4] | |
| [10105 | 149 | 7894 | 7] | |
| [870 | 15 | 12 | 134]] | |
|  | | | | |
| **Classification report** | | | | |
|  | **Precision** | **Recall** | **F1-score** | **Support** |
| 1 | 0.97 | 1.00 | 0.99 | 1583728 |
| 2 | 0.56 | 0.12 | 0.19 | 34420 |
| 3 | 0.84 | 0.43 | 0.57 | 18155 |
| 4 | 0.59 | 0.13 | 0.21 | 1031 |
|  | | | | |
| **Accuracy** |  |  | 0.97 | 1637334 |
| **Macro avg** | 0.74 | 0.42 | 0.49 | 1637334 |
| **Weighted avg** | 0.96 | 0.97 | 0.96 | 1637334 |

**Farm 4**

| **Image 1** | | | | | |
| --- | --- | --- | --- | --- | --- |
| **Confusion matrix** | | | | | |
| [[5775 | 18 | 0 | 23 | 62 | 325] |
| [60 | 717151 | 0 | 33821 | 429 | 313] |
| [0 | 148 | 65 | 889 | 0 | 0] |
| [5 | 83046 | 70 | 298765 | 298 | 147] |
| [93 | 1103 | 0 | 1225 | 40339 | 509] |
| [478 | 258 | 0 | 233 | 1132 | 9194]] |
|  | | | | | |
| **Classification report** | | | | | |
|  | **Precision** | **Recall** | **F1-score** | **Support** | |
| 1 | 0.90 | 0.93 | 0.92 | 6203 | |
| 2 | 0.89 | 0.95 | 0.92 | 751774 | |
| 3 | 0.48 | 0.06 | 0.11 | 1102 | |
| 4 | 0.89 | 0.78 | 0.83 | 382331 | |
| 5 | 0.95 | 0.93 | 0.94 | 43269 | |
| 6 | 0.88 | 0.81 | 0.84 | 11295 | |
|  | | | | | |
| **Accuracy** |  |  | 0.90 | 1195974 | |
| **Macro avg** | 0.83 | 0.75 | 0.76 | 1195974 | |
| **Weighted avg** | 0.90 | 0.90 | 0.89 | 1195974 | |

|  | | | | | |
| --- | --- | --- | --- | --- | --- |
| **Image 2** | | | | | |
| **Confusion matrix** | | | | | |
| [[15294 | 0 | 80 | 4 | 65 | 638] |
| [0 | 410514 | 4575 | 32364 | 53 | 632] |
| [186 | 19020 | 22217 | 21675 | 403 | 4257] |
| [8 | 67380 | 3465 | 297780 | 297 | 140] |
| [35 | 565 | 651 | 509 | 25062 | 276] |
| [757 | 1216 | 4002 | 195 | 347 | 18528]] |
|  | | | | | |
| **Classification report** | | | | | |
|  | **Precision** | **Recall** | **F1-score** | **Support** | |
| 1 | 0.94 | 0.95 | 0.95 | 16081 | |
| 2 | 0.82 | 0.92 | 0.87 | 448138 | |
| 3 | 0.63 | 0.33 | 0.43 | 67758 | |
| 4 | 0.84 | 0.81 | 0.83 | 369070 | |
| 5 | 0.96 | 0.92 | 0.94 | 27098 | |
| 6 | 0.76 | 0.74 | 0.75 | 25045 | |
|  | | | | | |
| **Accuracy** |  |  | 0.83 | 953190 | |
| **Macro avg** | 0.83 | 0.78 | 0.79 | 953190 | |
| **Weighted avg** | 0.82 | 0.83 | 0.82 | 953190 | |

| **Image 3** | | | | |
| --- | --- | --- | --- | --- |
| **Confusion matrix** | | | | |
| [[5952 | 124 | 5 | 2] | |
| [130 | 154742 | 2100 | 4] | |
| [1 | 4742 | 24851 | 0] | |
| [1 | 15 | 1 | 864]] | |
|  | | | | |
| **Classification report** | | | | |
|  | **Precision** | **Recall** | **F1-score** | **Support** |
| 1 | 0.98 | 0.98 | 0.98 | 6083 |
| 2 | 0.97 | 0.99 | 0.98 | 156976 |
| 4 | 0.92 | 0.84 | 0.88 | 29594 |
| 5 | 0.99 | 0.98 | 0.99 | 881 |
|  | | | | |
| **Accuracy** |  |  | 0.96 | 193534 |
| **Macro avg** | 0.97 | 0.95 | 0.96 | 193534 |
| **Weighted avg** | 0.96 | 0.96 | 0.96 | 193534 |

| **Image 4** | | | | |
| --- | --- | --- | --- | --- |
| **Confusion matrix** | | | | |
| [[56078 | 6171 | 95] | | |
| [6345 | 81334 | 97] | | |
| [133 | 41 | 3487]] | | |
|  | | | | |
| **Classification report** | | | | |
|  | **Precision** | **Recall** | **F1-score** | **Support** |
| 2 | 0.90 | 0.90 | 0.90 | 62344 |
| 4 | 0.93 | 0.93 | 0.93 | 87776 |
| 5 | 0.95 | 0.95 | 0.95 | 3661 |
|  | | | | |
| **Accuracy** |  |  | 0.92 | 153781 |
| **Macro avg** | 0.92 | 0.93 | 0.93 | 153781 |
| **Weighted avg** | 0.92 | 0.92 | 0.92 | 153781 |

**Farm 5**

| **Image 1** | | | | |
| --- | --- | --- | --- | --- |
| **Confusion matrix** | | | | |
| [[373717 | 9460 | 77 | 37 | 80] |
| [32748 | 53770 | 56 | 34 | 191] |
| [78 | 26 | 5478 | 203 | 0] |
| [16 | 14 | 59 | 18308 | 0] |
| [1404 | 1219 | 0 | 0 | 183] |
|  | | | | |
| **Classification report** | | | | |
|  | **Precision** | **Recall** | **F1-score** | **Support** |
| 1 | 0.92 | 0.97 | 0.94 | 383371 |
| 2 | 0.83 | 0.62 | 0.71 | 86799 |
| 3 | 0.97 | 0.95 | 0.96 | 5785 |
| 4 | 0.99 | 1.00 | 0.99 | 18397 |
| 5 | 0.40 | 0.07 | 0.11 | 2806 |
|  | | | | |
| **Accuracy** |  |  | 0.91 | 497158 |
| **Macro avg** | 0.82 | 0.72 | 0.74 | 497158 |
| **Weighted avg** | 0.90 | 0.91 | 0.90 | 497158 |

| **Image 2** | | | | |
| --- | --- | --- | --- | --- |
| **Confusion matrix** | | | | |
| [[767181 | 13601 | 136 | 41 | 7] |
| [117138 | 60452 | 49 | 71 | 22] |
| [5691 | 19 | 5064 | 188 | 0] |
| [2128 | 39 | 135 | 2493 | 0] |
| [1028 | 293 | 0 | 0 | 7] |
|  | | | | |
| **Classification report** | | | | |
|  | **Precision** | **Recall** | **F1-score** | **Support** |
| 1 | 0.86 | 0.98 | 0.92 | 780966 |
| 2 | 0.81 | 0.34 | 9.48 | 177732 |
| 3 | 0.94 | 0.46 | 0.62 | 10962 |
| 4 | 0.89 | 0.52 | 0.66 | 4795 |
| 5 | 0.19 | 0.01 | 0.01 | 1328 |
|  | | | | |
| **Accuracy** |  |  | 0.86 | 975783 |
| **Macro avg** | 0.74 | 0.46 | 0.54 | 975783 |
| **Weighted avg** | 0.85 | 0.86 | 0.83 | 975783 |

| **Image 3** | | | | |
| --- | --- | --- | --- | --- |
| **Confusion matrix** | | | | |
| [[1203987 | 23603 | 1020 | 95 | 37] |
| [104655 | 137911 | 204 | 62 | 116] |
| [4517 | 68 | 11964 | 667 | 0] |
| [334 | 42 | 703 | 5033 | 0] |
| [1645 | 1237 | 0 | 0 | 29]] |
|  | | | | |
| **Classification report** | | | | |
|  | **Precision** | **Recall** | **F1-score** | **Support** |
| 1 | 0.92 | 0.98 | 0.95 | 1228742 |
| 2 | 0.85 | 0.57 | 0.68 | 242948 |
| 3 | 0.86 | 0.69 | 0.77 | 17216 |
| 4 | 0.86 | 0.82 | 0.84 | 6112 |
| 5 | 0.16 | 0.01 | 0.02 | 2911 |
|  | | | | |
| **Accuracy** |  |  | 0.91 | 1497929 |
| **Macro avg** | 0.73 | 0.62 | 0.65 | 1497929 |
| **Weighted avg** | 0.90 | 0.91 | 0.90 | 1497929 |

| **Image 4** | | | | |
| --- | --- | --- | --- | --- |
| **Confusion matrix** | | | | |
| [[435674 | 14885 | 995 | 7 | 17] |
| [101391 | 95080 | 400 | 1 | 90] |
| [5194 | 1063 | 9904 | 651 | 1] |
| [5 | 1 | 692 | 3749 | 0] |
| [788 | 1020 | 14 | 0 | 13]] |
|  | | | | |
| **Classification report** | | | | |
|  | **Precision** | **Recall** | **F1-score** | **Support** |
| 1 | 0.80 | 0.96 | 0.88 | 451578 |
| 2 | 0.85 | 0.48 | 0.62 | 196962 |
| 3 | 0.82 | 0.59 | 0.69 | 16813 |
| 4 | 0.85 | 0.84 | 0.85 | 4447 |
| 5 | 0.11 | 0.01 | 0.01 | 1835 |
|  | | | | |
| **Accuracy** |  |  | 0.81 | 671635 |
| **Macro avg** | 0.69 | 0.58 | 0.61 | 671635 |
| **Weighted avg** | 0.81 | 0.81 | 0.79 | 671635 |

| **Image 5** | | | | |
| --- | --- | --- | --- | --- |
| **Confusion matrix** | | | | |
| [[710733 | 72954 | 147 | 13 | 14] |
| [59215 | 170696 | 28 | 48 | 40] |
| [87 | 5802 | 4935 | 425 | 0] |
| [6 | 31 | 254 | 5065 | 0] |
| [204 | 954 | 0 | 0 | 10]] |
|  | | | | |
| **Classification report** | | | | |
|  | **Precision** | **Recall** | **F1-score** | **Support** |
| 1 | 0.92 | 0.91 | 0.91 | 783861 |
| 2 | 0.68 | 0.74 | 0.71 | 230027 |
| 3 | 0.92 | 0.44 | 0.59 | 11249 |
| 4 | 0.91 | 0.95 | 0.93 | 5356 |
| 5 | 0.16 | 0.01 | 0.02 | 1168 |
|  | | | | |
| **Accuracy** |  |  | 0.86 | 1031661 |
| **Macro avg** | 0.72 | 0.61 | 0.63 | 1031661 |
| **Weighted avg** | 0.87 | 0.86 | 0.86 | 1031661 |

**Farm 6**

| **Image 1** | | | | |
| --- | --- | --- | --- | --- |
| **Confusion matrix** | | | | |
| [[302465 | 13831 | 1816 | 145 | 13] |
| [46720 | 50675 | 330 | 31 | 147] |
| [1400 | 188 | 27303 | 144 | 0] |
| [219 | 20 | 374 | 7171 | 0] |
| [606 | 1129 | 0 | 0 | 57]] |
|  | | | | |
| **Classification report** | | | | |
|  | **Precision** | **Recall** | **F1-score** | **Support** |
| 1 | 0.86 | 0.95 | 0.90 | 318270 |
| 2 | 0.77 | 0.52 | 0.62 | 97903 |
| 3 | 0.92 | 0.94 | 0.93 | 29035 |
| 4 | 0.96 | 0.92 | 0.94 | 7784 |
| 5 | 0.26 | 0.03 | 0.06 | 1792 |
|  | | | | |
| **Accuracy** |  |  | 0.85 | 454784 |
| **Macro avg** | 0.75 | 0.67 | 0.69 | 454784 |
| **Weighted avg** | 0.84 | 0.85 | 0.84 | 454784 |

| **Image 2** | | | | |
| --- | --- | --- | --- | --- |
| **Confusion matrix** | | | | |
| [[275751 | 12466 | 1136 | 99 | 23] |
| [22218 | 64524 | 0 | 2 | 1571] |
| [1189 | 2 | 5048 | 3 | 0] |
| [133 | 0 | 5 | 2631 | 0] |
| [736 | 4846 | 1 | 0 | 2502]] |
|  | | | | |
| **Classification report** | | | | |
|  | **Precision** | **Recall** | **F1-score** | **Support** |
| 1 | 0.92 | 0.95 | 0.94 | 289475 |
| 2 | 0.79 | 0.73 | 0.76 | 88315 |
| 3 | 0.82 | 0.81 | 0.81 | 6242 |
| 4 | 0.96 | 0.95 | 0.96 | 2769 |
| 5 | 0.61 | 0.31 | 0.41 | 8112 |
|  | | | | |
| **Accuracy** |  |  | 0.89 | 394913 |
| **Macro avg** | 0.82 | 0.75 | 0.77 | 394913 |
| **Weighted avg** | 0.88 | 0.89 | 0.88 | 394913 |

**Farm 7**

| **Confusion matrix** | | | | |
| --- | --- | --- | --- | --- |
| [[3034 | 17 | 0 | 2] | |
| [30 | 1267 | 3 | 0] | |
| [0 | 4 | 10 | 0] | |
| [8 | 0 | 0 | 30]] | |
|  | | | | |
| **Classification report** | | | | |
|  | **Precision** | **Recall** | **F1-score** | **Support** |
| 1 | 0.99 | 0.99 | 0.99 | 3053 |
| 2 | 0.98 | 0.97 | 0.98 | 1300 |
| 3 | 0.77 | 0.71 | 0.74 | 14 |
| 4 | 0.94 | 0.79 | 0.86 | 38 |
|  | | | | |
| **Accuracy** |  |  | 0.99 | 4405 |
| **Macro avg** | 0.92 | 0.87 | 0.89 | 4405 |
| **Weighted avg** | 0.99 | 0.99 | 0.99 | 4405 |
