# Supplementary material for: Epidemiological study of leptospiral interaction in bovine farms in rural areas of Colombia: A One Health approach
Source: PLoS Negl Trop Dis. 2026 May 6;20(5):e0014231. doi: 10.1371/journal.pntd.0014231 (PMC13170971; doi:10.1371/journal.pntd.0014231)
Supplement: S2 Table — (DOCX) [file pntd.0014231.s002.docx]

**S2 Table.** **Description of the landscape metrics for Farm 1.**

| **Land use cover class** | **Total area (ha)** | **Landscape proportion (%)** | **Number of patches** | **Patch density (patches per 100 ha)** | **Largest patch index (%)** | **Total edge (m)** | **Edge density (m/ha)** | **Landscape shape index** |
| --- | --- | --- | --- | --- | --- | --- | --- | --- |
| Pasture or forage | 105.03 | 78.85 | 3862 | 2899.48 | 77.00 | 14654.55 | 1097.28 | 36.82 |
| Forest or dense vegetation | 26.89 | 20.19 | 5300 | 3979.09 | 5.32 | 155578.95 | 1168.04 | 74.94 |
| Water bodies | 0.82 | 0.61 | 1837 | 1379.17 | 0.13 | 11439.18 | 85.88 | 31.38 |
| Built-up areas | 0.44 | 0.33 | 173 | 129.88 | 0.029 | 4018.28 | 30.16 | 14.95 |
| Crop cultivation | 0.0007 | 0.0005 | 5 | 3.75 | 0.0003 | 24.46 | 0.18 | 2.27 |
