# Supplementary material for: Epidemiological study of leptospiral interaction in bovine farms in rural areas of Colombia: A One Health approach
Source: PLoS Negl Trop Dis. 2026 May 6;20(5):e0014231. doi: 10.1371/journal.pntd.0014231 (PMC13170971; doi:10.1371/journal.pntd.0014231)
Supplement: S3 Table — (DOCX) [file pntd.0014231.s003.docx]

**S3 Table.** **Description of the landscape metrics for Farm 3.**

| **Land use cover class** | **Total area (ha)** | **Landscape proportion (%)** | **Number of patches** | **Patch density (patches per 100 ha)** | **Largest patch index (%)** | **Total edge (m)** | **Edge density (m/ha)** | **Landscape shape index** |
| --- | --- | --- | --- | --- | --- | --- | --- | --- |
| Pasture or forage | 233.94 | 88.20 | 6004 | 2263.80 | 87.06 | 270825.90 | 1021.14 | 46.19 |
| Forest or dense vegetation | 30.09 | 11.34 | 15705 | 5921.56 | 1.70 | 277036.15 | 1044.56 | 126.14 |
| Water bodies | 1.14 | 0.43 | 1258 | 474.32 | 0.028 | 12883.78 | 48.57 | 30.01 |
| Built-up areas | 0.028 | 0.01 | 17 | 6.40 | 0.004 | 274.45 | 1.03 | 4.08 |
