# Supplementary material for: Epidemiological study of leptospiral interaction in bovine farms in rural areas of Colombia: A One Health approach
Source: PLoS Negl Trop Dis. 2026 May 6;20(5):e0014231. doi: 10.1371/journal.pntd.0014231 (PMC13170971; doi:10.1371/journal.pntd.0014231)
Supplement: S4 Table — (DOCX) [file pntd.0014231.s004.docx]

**S4 Table.** **Description of the landscape metrics for Farm 4.**

| **Land use cover class** | **Total area (ha)** | **Landscape proportion (%)** | **Number of patches** | **Patch density (patches per 100 ha)** | **Largest patch index (%)** | **Total edge (m)** | **Edge density (m/ha)** | **Landscape shape index** |
| --- | --- | --- | --- | --- | --- | --- | --- | --- |
| Pasture or forage | 61.23 | 45.34 | 47 | 34.80 | 45.24 | 5726.83 | 42.40 | 3.18 |
| Forest or dense vegetation | 44.22 | 32.74 | 5566 | 4121.50 | 28.98 | 135918.03 | 1006.44 | 51.04 |
| Water bodies | 0.01 | 0.001 | 707 | 523.51 | 7.23E-05 | 382.50 | 2.83 | 25.58 |
| Built-up areas | 28.02 | 20.75 | 5474 | 4053.38 | 3.23 | 144106.69 | 1067.08 | 68.01 |
| Crop cultivation | 0.80 | 0.59 | 3852 | 2852.32 | 0.09 | 22176.64 | 164.21 | 61.44 |
