# Supplementary material for: Epidemiological study of leptospiral interaction in bovine farms in rural areas of Colombia: A One Health approach
Source: PLoS Negl Trop Dis. 2026 May 6;20(5):e0014231. doi: 10.1371/journal.pntd.0014231 (PMC13170971; doi:10.1371/journal.pntd.0014231)
Supplement: S5 Table — (DOCX) [file pntd.0014231.s005.docx]

**S5 Table.** **Description of the landscape metrics for Farm 5.**

| **Land use cover class** | **Total area (ha)** | **Landscape proportion (%)** | **Number of patches** | **Patch density (patches per 100 ha)** | **Largest patch index (%)** | **Total edge (m)** | **Edge density (m/ha)** | **Landscape shape index** |
| --- | --- | --- | --- | --- | --- | --- | --- | --- |
| Pasture or forage | 176.15 | 77.95 | 19251 | 8518.90 | 75.28 | 372824.66 | 1649.81 | 71.89 |
| Forest or dense vegetation | 47.16 | 20.87 | 20622 | 9125.59 | 2.76 | 396665.09 | 1755.31 | 144.02 |
| Water bodies | 1.84 | 0.81 | 4831 | 2137.80 | 0.01 | 33993.94 | 150.42 | 62.39 |
| Built-up areas | 0.81 | 0.35 | 490 | 216.83 | 0.04 | 6974.22 | 30.86 | 19.33 |
| Crop cultivation | 0.0001 | 5.33E-05 | 1 | 0.44 | 5.33E-05 | 5.20 | 0.02 | 1.17 |
