# Supplementary material for: Epidemiological study of leptospiral interaction in bovine farms in rural areas of Colombia: A One Health approach
Source: PLoS Negl Trop Dis. 2026 May 6;20(5):e0014231. doi: 10.1371/journal.pntd.0014231 (PMC13170971; doi:10.1371/journal.pntd.0014231)
Supplement: S6 Table — (DOCX) [file pntd.0014231.s006.docx]

**S6 Table. Description of the landscape metrics for Farm 6.**

| **Land use cover class** | **Total area (ha)** | **Landscape proportion (%)** | **Number of patches** | **Patch density (patches per 100 ha)** | **Largest patch index (%)** | **Total edge (m)** | **Edge density (m/ha)** | **Landscape shape index** |
| --- | --- | --- | --- | --- | --- | --- | --- | --- |
| Pasture or forage | 71.90 | 61.98 | 7120 | 6137.44 | 52.73 | 219559.78 | 1892.60 | 64.79 |
| Forest or dense vegetation | 42.90 | 36.98 | 9598 | 8273.48 | 12.28 | 222010.39 | 1913.73 | 87.50 |
| Water bodies | 0.80 | 0.69 | 1451 | 1250.76 | 0.23 | 10497.31 | 90.48 | 2.919.474.517 |
| Built-up areas | 0.36 | 0.31 | 250 | 215.50 | 0.03 | 4124.42 | 35.55 | 17.003 |
| Crop cultivation | 0.02 | 0.02 | 237 | 204.29 | 0.001 | 1033.97 | 8.91 | 16.52 |
