# Supplementary material for: Epidemiological study of leptospiral interaction in bovine farms in rural areas of Colombia: A One Health approach
Source: PLoS Negl Trop Dis. 2026 May 6;20(5):e0014231. doi: 10.1371/journal.pntd.0014231 (PMC13170971; doi:10.1371/journal.pntd.0014231)
Supplement: S7 Table — (DOCX) [file pntd.0014231.s007.docx]

**S7 Table. Description of the landscape metrics for Farm 7.**

| **Land use cover class** | **Total area (ha)** | **Landscape proportion (%)** | **Number of patches** | **Patch density (patches per 100 ha)** | **Largest patch index (%)** | **Total edge (m)** | **Edge density (m/ha)** | **Landscape shape index** |
| --- | --- | --- | --- | --- | --- | --- | --- | --- |
| Pasture or forage | 82.16 | 74.42 | 51 | 46.19 | 53.03 | 47914.08 | 434.00 | 15.06 |
| Forest or dense vegetation | 27.41 | 24.83 | 392 | 355.07 | 2.02 | 47116.39 | 426.77 | 22.98 |
| Water bodies | 0.07 | 0.07 | 25 | 22.64 | 0.04 | 466.99 | 4.23 | 4.18 |
| Built-up areas | 0.74 | 0.67 | 56 | 50.72 | 0.06 | 2817.31 | 25.51 | 8.18 |
