# Supplementary material for: Epidemiological study of leptospiral interaction in bovine farms in rural areas of Colombia: A One Health approach
Source: PLoS Negl Trop Dis. 2026 May 6;20(5):e0014231. doi: 10.1371/journal.pntd.0014231 (PMC13170971; doi:10.1371/journal.pntd.0014231)

**S5 Fig. Orthomosaic and land classification of the Farm 6.**

(A) Orthomosaic of the Farm 6. (B) Land classification of the Farm 6, showing: pastures (light green), dense vegetation (dark green), water bodies (blue) and built-up areas (red) (automatic classification using Python version 3.9). The orthomosaic was created by manually delineating the farm boundaries using an unmanned aerial vehicle to collect the data, and the analysis was performed using Agisoft Metashape 1.8 software. This figure is an original output generated by the authors and contains no third-party copyrighted material.


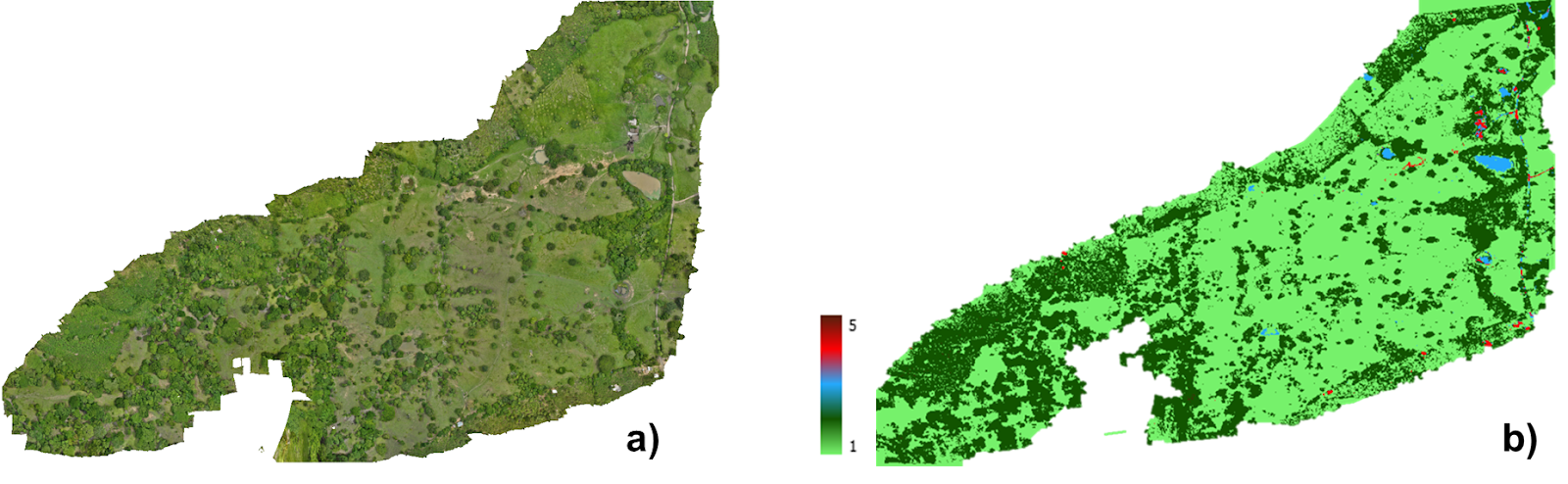

Supplement: S5 Fig — (A) Orthomosaic of the Farm 6. (B) Land classification of the Farm 6, showing: pastures (light green), dense vegetation (dark green), water bodies (blue) and built-up areas (red). The base map was developed through the manual delineation of farm boundaries by the research team. The map was created in QGIS software. (DOCX) [file pntd.0014231.s013.docx]
