# Supplementary material for: Epidemiological study of leptospiral interaction in bovine farms in rural areas of Colombia: A One Health approach
Source: PLoS Negl Trop Dis. 2026 May 6;20(5):e0014231. doi: 10.1371/journal.pntd.0014231 (PMC13170971; doi:10.1371/journal.pntd.0014231)

**S6 Fig. Orthomosaic and land classification of the Farm 7.**

(A) Orthomosaic of the Farm 7. (B) Land classification of the Farm 7, showing: pastures (light green), dense vegetation (dark green), water bodies (blue) and built-up areas (red) (automatic classification using Python version 3.9). The orthomosaic was generated using layers from QGIS software, which were obtained from publicly available sources compatible with CC BY 4.0 licensing.


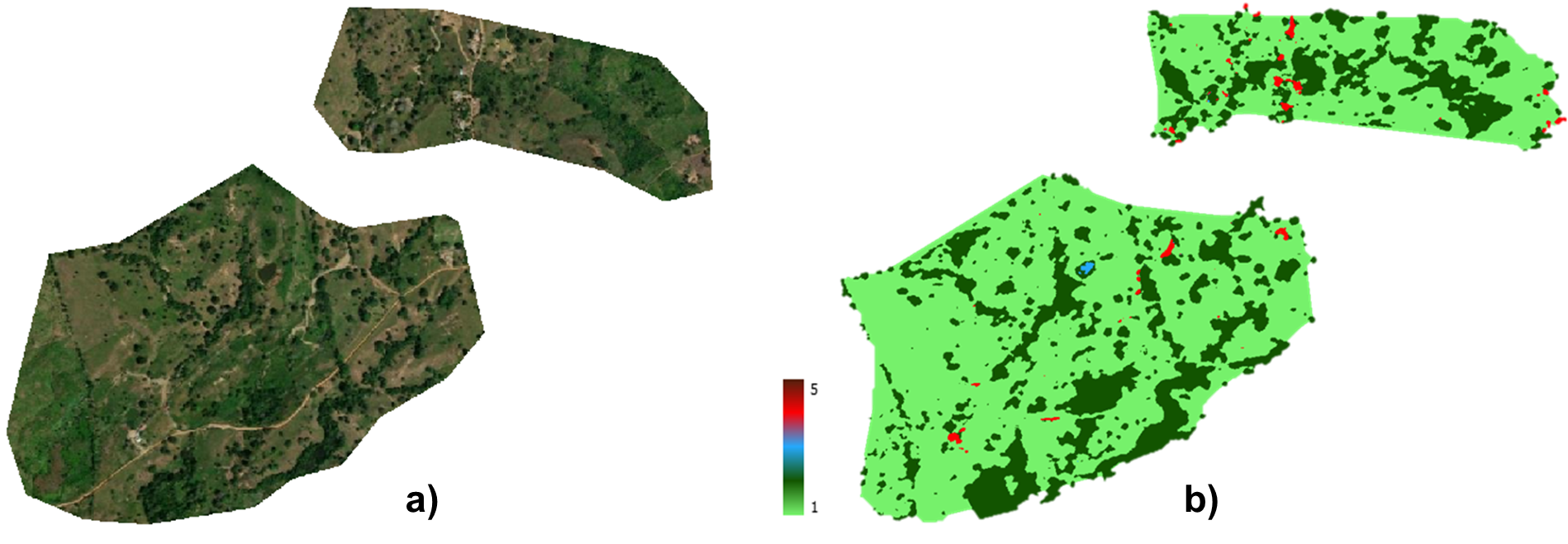

Supplement: S6 Fig — (A) Orthomosaic of the Farm 7. (B) Land classification of the Farm 7, showing: pastures (light green), dense vegetation (dark green), water bodies (blue) and built-up areas (red). The base map was developed through the manual delineation of farm boundaries by the research team. The map was created in QGIS software. (DOCX) [file pntd.0014231.s014.docx]
