# Supplementary material for: Epidemiological study of leptospiral interaction in bovine farms in rural areas of Colombia: A One Health approach
Source: PLoS Negl Trop Dis. 2026 May 6;20(5):e0014231. doi: 10.1371/journal.pntd.0014231 (PMC13170971; doi:10.1371/journal.pntd.0014231)

**S12 Fig. Digital elevation model and flooded area of the Farm 7.**

(A) Digital Elevation Model (DEM) of the Farm 7. (B) Flooded area of the Farm 7. (C) Proportion of floodable area of the Farm 7. The DEM, flooded area and proportions of floodable were generated using QGIS software and obtained from publicly available sources that are compatible with CC BY 4.0 licensing.


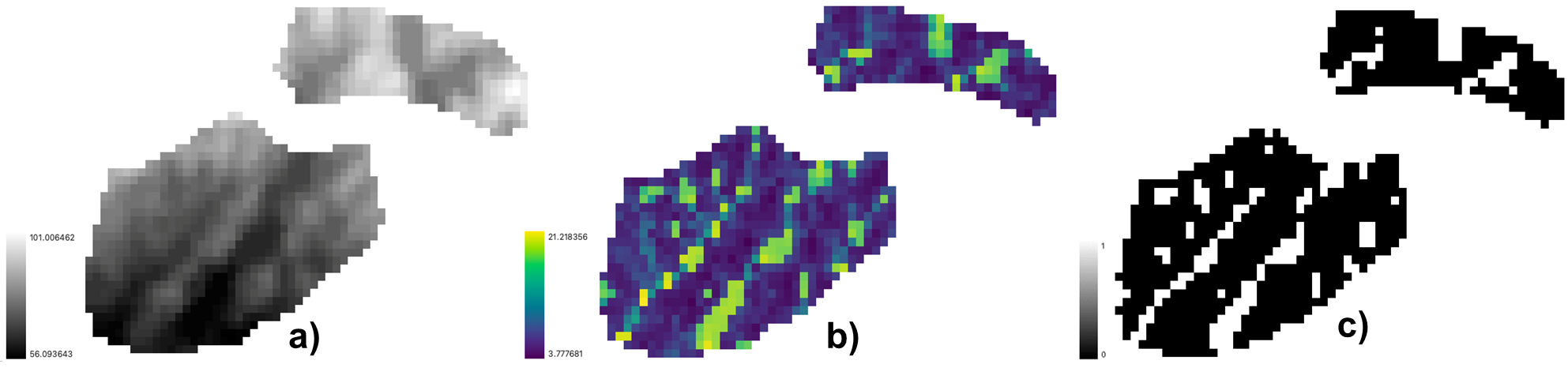

Supplement: S12 Fig — (A) Digital elevation model of the Farm 7. (B) Flooded area of the Farm 7. (C) Proportion of floodable area of the Farm 7. The base map was developed through the manual delineation of farm boundaries by the research team. The map was created in QGIS software. (DOCX) [file pntd.0014231.s020.docx]
