# Supplementary material for: Epidemiological study of leptospiral interaction in bovine farms in rural areas of Colombia: A One Health approach
Source: PLoS Negl Trop Dis. 2026 May 6;20(5):e0014231. doi: 10.1371/journal.pntd.0014231 (PMC13170971; doi:10.1371/journal.pntd.0014231)

**S13 Fig: Contribution of qualitative variables to dimension 1 in the MDFA.**

***Si_can****: presence of canines in paddocks,* ***P1_A****: contamination by Leptospira of subclade P1 in water sources,* ***MA_longB****: very high edge length,* ***MA_frag****: very high fragmentation,* ***MA_den_P****: very high patch density,* ***F_Mcomp****: very complex landscape shape,* ***Si_ro****: presence of rodents in paddocks,* ***F_comp****: complex landscape shape,* ***A_longB****: high edge length,* ***A_frag****: high fragmentation,* ***A_den_P****: high patch density,* ***Sej_per****: seropositivity to Sejroe serogroup in humans,* ***No_can****: absence of canines in paddocks,* ***A_den_B****: high edge density,* ***NA_pers****: seronegativity to Leptospira in humans,* ***MA_den_B****: very high edge density,* ***Neg_A****: water with no detection of pathogenic Leptospira contamination,* ***B_rep****: low representation of dense vegetation area in the landscape,* ***<7****: paddock rotation every 7 days,* ***MX_prr****: mixed seropositivity in canines,* ***Ict_pers****: seropositivity to Icterohaemorrhagiae serogroup in humans,* ***<3****: paddock rotation every 3 days,* ***M_peq****: very small dense vegetation area,* ***M_rep****: medium representation of dense vegetation area in the landscape,* ***1_EA****: < 1 year of age in cattle.*


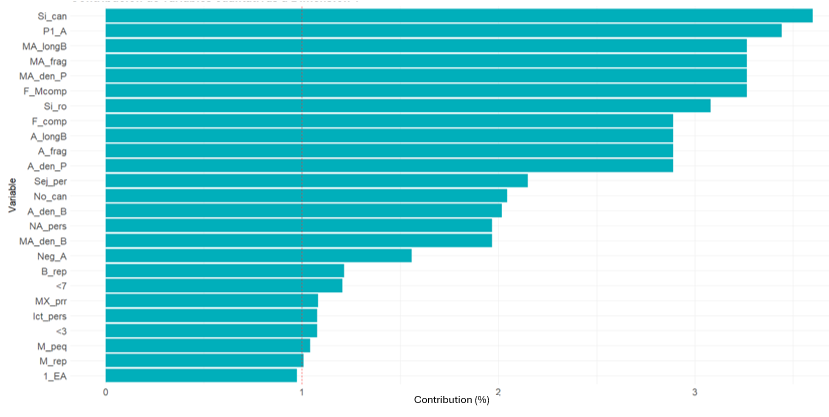

Supplement: S13 Fig — Si_can: presence of canines in paddocks, P1_A: contamination by Leptospira of subclade P1 in water sources, MA_longB: very high edge length, MA_frag: very high fragmentation, MA_den_P: very high patch density, F_Mcomp: very complex landscape shape, Si_ro: presence of rodents in paddocks, F_comp: complex landscape shape, A_longB: high edge length, A_frag: high fragmentation, A_den_P: high patch density, Sej_per: seropositivity to Sejroe serogroup in humans, No_can: absence of canines in paddocks, A_den_B: high edge density, NA_pers: seronegativity to Leptospira in humans, MA_den_B: very high edge density, Neg_A: water with no detection of pathogenic Leptospira contamination, B_rep: low representation of dense vegetation area in the landscape, < 7: paddock rotation every 7 days, MX_prr: mixed seropositivity in canines, Ict_pers: seropositivity to Icterohaemorrhagiae serogroup in humans, < 3: paddock rotation every 3 days, M_peq: very small dense vegetation area, M_rep: medium representation of dense vegetation area in the landscape, 1_EA: < 1 year of age in cattle. (DOCX) [file pntd.0014231.s021.docx]
