# Supplementary material for: Epidemiological study of leptospiral interaction in bovine farms in rural areas of Colombia: A One Health approach
Source: PLoS Negl Trop Dis. 2026 May 6;20(5):e0014231. doi: 10.1371/journal.pntd.0014231 (PMC13170971; doi:10.1371/journal.pntd.0014231)

**S14 Fig: Contribution of qualitative variables to dimension 2 in the MDFA.**

***Frag****: largest fragmented patch,* ***Ict_pers****: seropositivity to Icterohaemorrhagiae serogroup in humans,* ***<3****: paddock rotation every 3 days,* ***M_peq****: very small dense vegetation area,* ***A_inun****: highly floodable landscape,* ***M_frag****: largest highly fragmented patch,* ***Peq****: small dense vegetation area,* ***MA_inun****: very highly floodable landscape,* ***B_rep****: low representation of dense vegetation area in the landscape,* ***M_rep****: medium representation of dense vegetation area in the landscape,* ***Sej_per****: seropositivity to Sejroe serogroup in humans,* ***Ict_prr****: seropositivity to Icterohaemorrhagiae serogroup in canines,* ***HL****:growing heifer,* ***Si_V****: vaccination against Leptospira in cattle,* ***P1_P2_S****: contamination by Leptospira of subclades P1 and P2 in soils,* ***2_EA****: > 1 to 4 years of age in cattle,* ***<7****: paddock rotation every 7 days,* ***Neg_prr****: seronegativity to Leptospira in canines,* ***MX_prr****: mixed seropositivity in canines,* ***1_EA****: < 1 year of age in cattle,* ***Can_Aut****: seropositivity to Autumnalis and Canicola serogroups in canines,* ***Aut_pers****: seropositivity to Autumnalis serogroup in humans,* ***CM****: bull calf,* ***L_Borg****: infection by L. borgpetersenii in cattle,* ***CH****: heifer calf.*


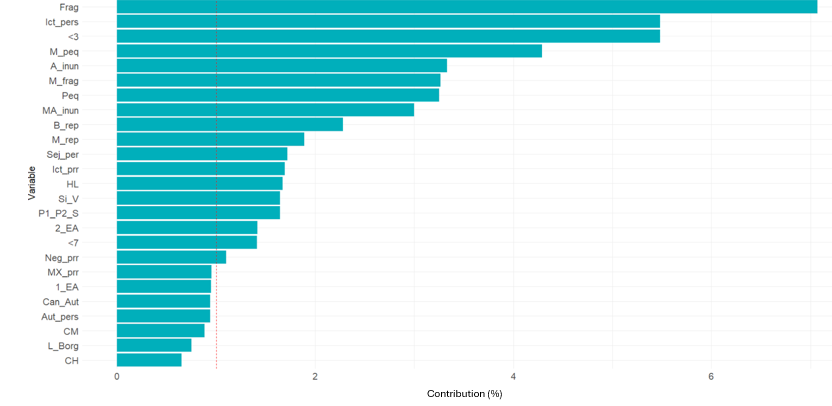

Supplement: S14 Fig — Frag: largest fragmented patch, Ict_pers: seropositivity to Icterohaemorrhagiae serogroup in humans, < 3: paddock rotation every 3 days, M_peq: very small dense vegetation area, A_inun: highly floodable landscape, M_frag: largest highly fragmented patch, Peq: small dense vegetation area, MA_inun: very highly floodable landscape, B_rep: low representation of dense vegetation area in the landscape, M_rep: medium representation of dense vegetation area in the landscape, Sej_per: seropositivity to Sejroe serogroup in humans, Ict_prr: seropositivity to Icterohaemorrhagiae serogroup in canines, HL:growing heifer, Si_V: vaccination against Leptospira in cattle, P1_P2_S: contamination by Leptospira of subclades P1 and P2 in soils, 2_EA: > 1–4 years of age in cattle, < 7: paddock rotation every 7 days, Neg_prr: seronegativity to Leptospira in canines, MX_prr: mixed seropositivity in canines, 1_EA: < 1 year of age in cattle, Can_Aut: seropositivity to Autumnalis and Canicola serogroups in canines, Aut_pers: seropositivity to Autumnalis serogroup in humans, CM: bull calf, L_Borg: infection by L. borgpetersenii in cattle, CH: heifer calf. (DOCX) [file pntd.0014231.s022.docx]
