# Supplementary material for: Epidemiological study of leptospiral interaction in bovine farms in rural areas of Colombia: A One Health approach
Source: PLoS Negl Trop Dis. 2026 May 6;20(5):e0014231. doi: 10.1371/journal.pntd.0014231 (PMC13170971; doi:10.1371/journal.pntd.0014231)

**S15 Fig: Contribution of quantitative variables to dimension 1 in the MDFA.**

***tot_edge:*** *total edge,* ***pat_dens:*** *patch density,* ***lnd_shap:*** *landscape shape index,* ***n_patch:*** *number of patches,* ***edg_dens:*** *edge density,* ***tot_area:*** *total dense vegetation area,* ***pro_pers:*** *proportion of seropositivity in humans,* ***lar_p_ix:*** *largest patch index,* ***prp_land:*** *proportion of dense vegetation area within the landscape,* ***ith:*** *topographic wetness index.*


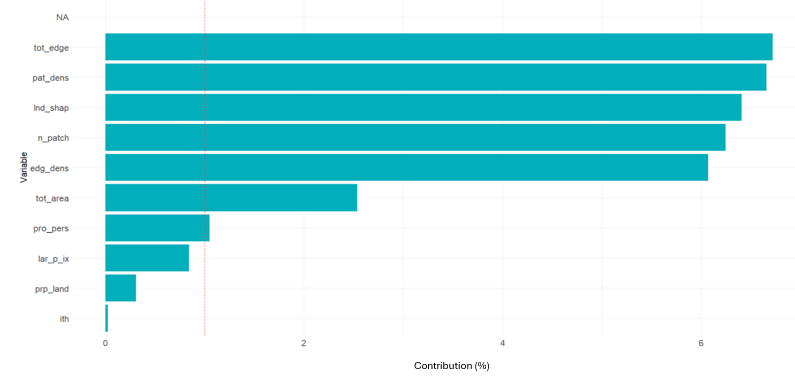

Supplement: S15 Fig — tot_edge: total edge, pat_dens: patch density, lnd_shap: landscape shape index, n_patch: number of patches, edg_dens: edge density, tot_area: total dense vegetation area, pro_pers: proportion of seropositivity in humans, lar_p_ix: largest patch index, prp_land: proportion of dense vegetation area within the landscape, ith: topographic wetness index. (DOCX) [file pntd.0014231.s023.docx]
