# Supplementary material for: Epidemiological study of leptospiral interaction in bovine farms in rural areas of Colombia: A One Health approach
Source: PLoS Negl Trop Dis. 2026 May 6;20(5):e0014231. doi: 10.1371/journal.pntd.0014231 (PMC13170971; doi:10.1371/journal.pntd.0014231)
Supplement: S16 Fig — lar_p_ix: largest patch index, ith: topographic wetness index, prp_land: proportion of dense vegetation area within the landscape, tot_area: total dense vegetation area, pro_pers: proportion of seropositivity in humans, edg_dens: edge density, pat_dens: patch density, lnd_shap: landscape shape index,n_patch: number of patches, tot_edge: total edge. (DOCX) [file pntd.0014231.s024.docx]

**S16 Fig: Contribution of quantitative variables to dimension 2 in the MDFA.**

***lar_p_ix:*** *largest patch index,* ***ith:*** *topographic wetness index,* ***prp_land:*** *proportion of dense vegetation area within the landscape,* ***tot_area:*** *total dense vegetation area,* ***pro_pers:*** *proportion of seropositivity in humans,* ***edg_dens:*** *edge density,* ***pat_dens:*** *patch density,* ***lnd_shap:*** *landscape shape index,****n_patch:*** *number of patches,* ***tot_edge:*** *total edge.*

*
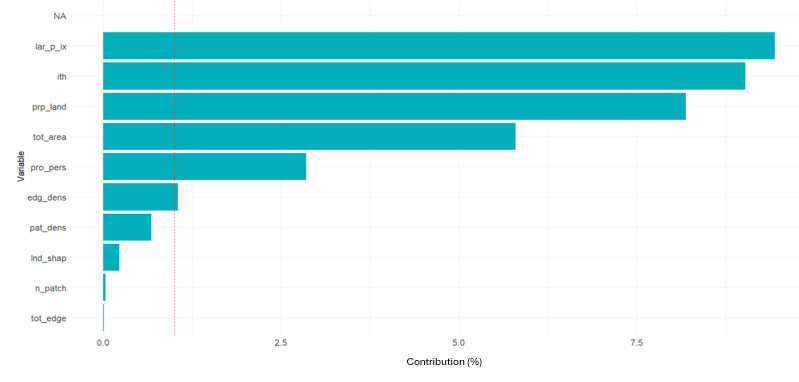
*
